# Production scheme for diagnostic-therapeutic radioisotopes by accelerator neutrons

**DOI:** 10.2183/pjab.97.017

**Published:** 2021-06-11

**Authors:** Yasuki NAGAI

**Affiliations:** *1 Professor Emeritus, Osaka University, Suita, Osaka, Japan.; *2 Professor Emeritus, Tokyo Institute of Technology, Tokyo, Japan.; *3 QST-Associate, National Institute for Quantum and Radiological Science and Technology, Tokai, Ibaraki, Japan.; *4 Research Professor, Tohoku University, Sendai, Miyagi, Japan.; *5 Research Fellow, Chiyoda Technol Co., Tokyo, Japan.

**Keywords:** radioisotopes, radiopharmaceuticals, diagnosis, therapy, deuteron accelerator, accelerator neutrons

## Abstract

Interest has been growing in the development of medical radioisotopes used for noninvasive nuclear medicine imaging of disease and cancer therapy. Especially the development of an alternative production scheme of ^99^Mo, the mother radioisotope of ^99m^Tc used for imaging, is required, because the current supply chain of the reactor product ^99^Mo is fragile worldwide. We have proposed a new production scheme of ^99^Mo as well as therapeutic radioisotopes, such as ^64^Cu and ^67^Cu, using accelerator neutrons provided by the ^nat^C(*d*,*n*) reaction. Based on this scheme we have obtained high-quality ^99m^Tc, ^64^Cu, and ^67^Cu suitable for clinical use by developing both production and separation methods of the radioisotopes. We proposed a new facility to constantly and reliably produce a wide variety of high-quality, carrier-free radioisotopes, including ^99^Mo, with accelerator neutrons. We report on the development of the proposed scheme and future prospects of the facility toward the domestic production of medical radioisotopes.

## 1 Introduction

More than 150 different radioisotopes (RIs) have been used in medical, industrial, research as well as commercial applications, such as food processing, agriculture, and structural safety.^1)^ In these applications, the radioindicator tracer method, which was discovered by George de Hevesy, has been playing an important role.

### 1.1 Discovery of a radioindicator tracer concept.

George de Hevesy, known as the founder of radioanalytical chemistry and nuclear medicine, published a paper in 1913 claiming that radioindicator tracers play a unique role in chemical analysis.^2)^ The concept of radioindicator tracer chemistry was born through a talk with Ernest Rutherford.^3)^ He encouraged Hevesy to separate the natural radioisotope of lead-210 (^210^Pb) from its admixture with large amounts of nonradioactive lead stored at that time in Rutherford’s laboratory. Hevesy used chemical methods for such separation. After failing at this, Hevesy had an idea to use ^210^Pb as an indicator of lead. Hevesy studied the transfer of lead from soil in different parts of bean plants using ^210^Pb, which was the first application of the radioactive tracer technique to biology.^3)^ The extreme sensitivity of physical radioassay methods allowed him to carry out these experiments with such minuscule concentrations of lead so as to avoid its toxic properties. In 1935 Hevesy investigated the distribution and kinetics of the exchange of phosphorus in different parts of animals using the ^32^P radioisotope, which was the first radioindicator study in life sciences.^3)^

The essence of the radioindicator tracer method is that radioactivity is of such minute quantities that it will not cause any toxic affect on the system, and can be detected with high sensitivity by discriminating any conceivable background events as low as possible. A radiolabeled tracer allows noninvasive measurements of the distribution and function in a biological system. Thus, a door to so far unexplored nuclear medicine had been opened.

### 1.2 Medical radioisotopes for diagnostics and therapy in nuclear medicine.

Medical RIs in the form of radiopharmaceuticals have been used for noninvasive diagnostic imaging studies of diseases and therapeutic applications in cancer treatments, resulting to account for the majority of the applications of RIs.^1)^ Recently, an approach involving the merging of therapeutic and diagnosis (imaging) treatments, called the “theranostics” approach, has become very important to make a personalized medicine treatment for a specific patient.^4)^ Personalized medicine is a form of medicine that uses information about a patient’s own genes or proteins to prevent, diagnose, or treat some disease. A personalized medicine treatment in nuclear medicine is being made using theranostic radioisotopes in the same patients as follows. Firstly low-dose molecular imaging is performed to obtain necessary pre-treatment information concerning the patient’s biodistribution and dosimetry in patients, and secondly higher-dose target molecules therapy is carried out.^5)^ In order to promote this theranostics approach while aiming at personalized medicine treatment, the development of a variety of medical radioisotopes for the imaging and therapy of various diseases (especially cancers) is essential. The term “theranostics” was coined by Funkhouser.^6)^

Radioisotope imaging is divided into two modalities based on the type of decay and resultant particle emission and detection.^4)^ Single-photon emission-computed tomography (SPECT) is performed using γ-ray-emitting radioisotopes with energy below about 400 keV, which can penetrate the patient’s body and be detected by a gamma camera. Technetium-99m (^99m^Tc) with a half-life (*T*_1/2_ = 6 h) is the most widely used radioisotope in diagnostic imaging studies with SPECT.^4)^ Positron emission tomography (PET) imaging is the second-most common imaging modality with the use of a positron (β^+^)-emitting radioisotope. Fluorine-18 (^18^F) with *T*_1/2_ = 1.8 h has excellent nuclear properties for a PET radioisotope to provide optimal resolution, and an appropriate half-life for making radiopharmaceuticals.^4)^

Cancer treatments have been achieved by performing surgery, chemotherapy, radiation therapy, immunotherapy, targeted therapy, and hormone therapy *etc.* Radiation therapy is carried out using a certain amount of radiation doses to kill cancer cells and shrink tumors. It is divided into external radiation therapy, which is performed using such particles as X-rays, electrons, protons, and carbons, and internal radiation therapy which is carried out using therapeutic radiopharmaceuticals, including radioisotopes and pharmaceuticals, by employing techniques involving either brachytherapy or radioimmunotherapy.^5)^ In brachytherapy, radioisotopes are placed inside or near tumors of patients to treat cancer cells. Note that growing research activities include combining radiopharmaceuticals with conventional treatments, such as chemotherapy and external beam radiotherapy, that would need a variety of medical RIs to form effective radiopharmaceuticals.

Medical RIs used for internal radiation therapy should have the following physical and chemical properties^4)^: firstly, physical properties, such as the physical half-life (*T*_1/2_), particle decay mode (β^±^-ray, Auger electron, γ-ray, α-particle emission), and the energy of the emitted particle are important. The half-life of RIs must match with the pharmacokinetics of the radioactive drug for uptake and clearance from normal versus targeted (disease site) tissues. It is a crucial point for any radiopharmaceuticals to maximize the dose to the target tissue and minimize the dose to normal tissue. An excellent medical radioisotope used for diagnostics and therapeutics has a short half-life, typically within several hours, like ^18^F, and within ten days, respectively. Secondly, the chemical properties should be suitable for allowing the radioisotope to be incorporated into all sorts of molecules, including such as protein compounds for labeling the RIs with pharmaceuticals having high radiochemical yields and a high radionuclide purity. Thirdly, we have to prepare carrier-free RIs (without any other isotope of a sample nuclide) with high specific activity, because a typical activity of a ^99m^Tc radiopharmaceuticals solution administered to a patient is as high as about 740 MBq/(a few ml).^4)^ Note that the specific activity, which is defined as the activity per quantity of the atoms of a particular radionuclide, is usually given in units of Bq/g.

### 1.3 Production of medical radioisotopes in reactors and accelerators.

Most of medical radioisotopes are produced in reactors or accelerators. In reactors neutron-rich RIs are produced via the fission reaction of ^235^U or the thermal neutron capture reaction by a sample nucleus. The specific activity of RIs produced by the former reaction is expected to be high because carrier-free RIs can be obtained by a chemical separation process owing to the fact that an atomic number of the RIs differs from that of ^235^U. The specific activity of the RIs produced by the latter reaction is mostly very low because an atomic number of the RIs is the same as that of a sample, and therefore the RIs are not possible to be chemically separated from the sample. Neutron-rich nuclei decay by emitting β^−^-rays and γ-rays, and therefore radiopharmaceuticals containing β^−^-ray emitting RIs are used for radioimmunotherapy (RIT), and those containing γ-ray emitting RIs are used to diagnose the dynamics in a living body via SPECT. In accelerators, a variety of carrier-free RIs including many proton-rich light RIs, such as ^18^F and ^11^C, are produced using mostly proton beams.^5)^ These RIs decay mostly by emitting β^+^-rays or alpha particle, which are used in imaging and RIT, respectively. Typical medical RIs produced in reactors and accelerators are listed in Table 1.^7)^

### 1.4 Challenges in developing medical radioisotopes.

Currently there are important challenges in the productions of medical RIs used for diagnosis and therapy, respectively. Firstly, the supply chain of ^99^Mo (*T*_1/2_ = 66 h) is vulnerable and unreliable, which has frequently caused a shortage of ^99^Mo worldwide since 2008.^8)^ More than 80% of all diagnostic procedures in the world are carried out using ^99m^Tc obtained from a ^99^Mo/^99m^Tc generator. In Japan approximately 0.9 million procedures/year (about 2,750 procedures/day) are performed using ^99m^Tc.^9)^ Japan imports all of its required ^99^Mo several times per week. Therefore, a reliable and constant supply of ^99^Mo is a key issue for ensuring the routine application of ^99m^Tc worldwide. About 95% of ^99^Mo is produced by the fission reaction of enriched ^235^U in several aging research reactors around the world. The vulnerable situation of the supply chain of ^99^Mo is the impetus to study alternative methods for producing ^99^Mo and/or ^99m^Tc in reactors or accelerators worldwide.^8)^ In fact, strong efforts have been undertaken to develop an alternative production route of ^99^Mo. Until now, as far as we know, no alternative method has yet succeeded to secure a constant supply of ^99^Mo. Secondly, in order to promote a theranostics approach aimed at a personalized medicine treatment for a specific patient, the development of theranostic radioisotopes is highly required. Recent great success in the development of therapeutic RI of ^177^Lu (in reactors)^10)^ and ^225^Ac (decay product of ^229^Th of nuclear waste in reactors)^11)^ has been prompting further development of a wide variety of medical RIs by innovative production methods.

This review focuses on our studies, started in 2010, that aim to develop a new scheme of medical radioisotopes production and separation to secure a reliable supply chain of ^99^Mo for domestic use and to proceed the theranostics approach while aiming at personalized nuclear medicine. We discuss ^99m^Tc imaging and the supply chain of ^99^Mo in chapter 2. The new production scheme of medical radioisotopes, such as ^99^Mo, ^64^Cu and ^67^Cu, using accelerator neutrons is introduced in chapter 3. The development of separation methods of ^99m^Tc from ^99^Mo, and ^64^Cu and ^67^Cu from Zn samples are given in chapter 4. The deuteron accelerator and the accelerator neutron source are described in chapter 5. Conclusions and future prospects are given in chapter 6.

## 2 ^99m^Tc diagnostic imaging and ^99^Mo supply chain

**Figure 1.**
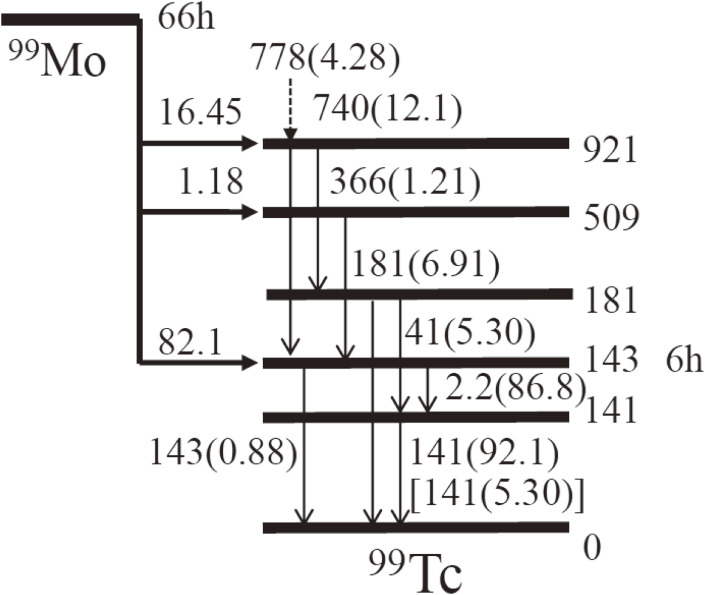
Partial decay scheme of ^99^Mo (*T*_1/2_ = 66 h) to ^99m^Tc (*T*_1/2_ = 6 h). Parenthesis: transition intensity (%). Parenthesis in square brackets: transition intensity (%) from the 181 keV state to the 141 keV one. (2004) Table of Radioactive Isotopes. Version 2.1. available on http://ie.lbl.gov/toi/index.asp (Ref. 12).

The excited state of ^99m^Tc at 143 keV is populated by the decay of ^99^Mo, as shown in Fig. 1.^12)^ ^99m^Tc has physical and chemical properties suitable to perform a diagnostic procedure.^4)^ First, a short half-life of ^99m^Tc (*T*_1/2_ = 6 h) allows one to use a large quantity of ^99m^Tc activity with a low radiation dose to a patient for obtaining clear imaging. Second, 141 keV γ-rays emitted from ^99m^Tc to the ground state of ^99^Tc are detected by a gamma camera with a high detection efficiency with a low-energy collimator. Third, ^99m^Tc has a versatile chemistry that allows it to be incorporated into all sorts of molecules. Fourth, ^99m^Tc is routinely produced in ^99^Mo/^99m^Tc generators over a period of about one week.

### 2.1 Discoveries of Tc and a Mo/Tc generator.

Tc is an unstable element. In 1937, an artificial element, Tc (^99m^Tc), was discovered by E. Segrè and C. Perrier in a sample obtained by bombarding a natural Mo sample with deuterons in the 37-inch cyclotron of the Berkeley Radiation Laboratory (now Lawrence Berkeley National Laboratory).^13)^ A radioactive Mo species (*T*_1/2_ = 66 h) was obtained, identified as ^99m^Tc.^14)^ Note that a cyclotron was invented by L. O. Lawrence in 1929–1930.^15)^ In order to separate element-43 from Mo metal a thermo-separation (sublimation) method, discussed later, was first employed by Perrier and Segrè.

A ^99^Mo/^99m^Tc generator, currently used to elute ^99m^Tc from ^99^Mo, was developed by W. Tucker and M. Greene at Brookhaven National Laboratory in 1958.^16)^ When they developed a system to separate ^132^I (*T*_1/2_ = 77 h) from its parent ^132^Te (*T*_1/2_ = 2.3 h), aiming at medical studies, they happened to detect a trace contaminant, which was later proved to be ^99m^Tc. It was then realized that ^99m^Tc was coming from its parent, ^99^Mo. Similarities between the chemistry of the tellurium-iodine parent-daughter pair and the molybdenum-technetium pair led to the development of a ^99^Mo/^99m^Tc generator system.

Note that Tc, presumably ^99^Tc with a relatively short half-life of 2.1 × 10^5^ y, compared to the age of the universe of 13.7 billion years, was discovered by P. W. Merrill at the surface of red giant stars in 1952.^17)^ The observation of Tc provided the first powerful evidence that Tc has been synthesized recently within stars. This was a great contribution for understanding the origins of elements heavier than iron observed in stars.

### 2.2 Production of ^99^Mo and supply chain of ^99^Mo.

About 95% of ^99^Mo is produced by the fission reaction of enriched ^235^U [hereafter: fission-^99^Mo] in a limited number of aging research reactors. In around 2008, just before the shortage of ^99^Mo began, five aging nuclear research reactors in The Netherlands, Australia, South Africa, Belgium, and France contributed to meet nearly all (about 95%) of the world’s supply of ^99^Mo/^99m^Tc.^8)^ Between 2008 and 2010 a supply crisis of ^99^Mo, caused by repeated shutdowns, resulted in many diagnostic tests being cancelled or delayed.^8)^ The incidents of the reactors highlighted shortcomings and unreliability in the supply of ^99m^Tc. Currently, ^99^Mo has been mostly produced in seven research reactors, listed in Table 2.^18,19)^

**Figure 2.**
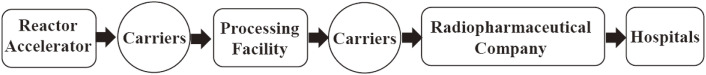
Schematic view of the supply chain of ^99^Mo from producers to users. At a processing facility ^99^Mo is chemically separated and purified from a fission product of ^235^U. IAEA, Belgium, INIS-BE-10K0001, 2010 (Ref. 20).

In addition to concerns related to aging reactors, it is worth noting that the global supply chain of ^99^Mo for the ^99^Mo/^99m^Tc generator production is complex, as shown in Fig. 2.^20)^ Namely, after being produced in reactors, ^99^Mo is transferred to a processing facility (as listed in Table 3) to be chemically separated and purified. The finished ^99^Mo product material is then isolated and shipped to one of eight generator-manufacturing facilities, located in different countries, that supply ^99^Mo in the form of a ^99^Mo/^99m^Tc generator to end users, such as nuclear pharmacies and hospitals. In addition, the time frame to deliver purified ^99^Mo to generator manufacturers is limited because of the short half-life of ^99^Mo (*T*_1/2_ = 66 h). These complex supply chains would cause transportation obstacles, such as customs, government regulations, flight schedules, weather delays, and pilot refusal. Natural disasters also have the potential to result in significant product shipment delays. A volcano that erupted in Iceland also reminded us of the risk of relying on a small number of ^99^Mo production facilities in the world. There are currently only five ^99^Mo-processing facilities in the world, as listed in Table 3, in which three of them will end their operations within a decade.^18,19)^

### 2.3 Action by Organisation for Economic Cooperation and Development (OECD).

Under such a circumstance of fragility of the ^99^Mo supply chain, the OECD Nuclear Energy Agency (NEA) has started global efforts to ensure reliable supplies of ^99^Mo and ^99m^Tc.^21)^ The NEA Steering Committee for Nuclear Energy established the High-level Group on the Security of Supply of Medical Radioisotopes (HLG-MR) in 2009 to strengthen the reliability of ^99^Mo and ^99m^Tc supply in both the short (between 2010 and 2017), medium (2017–2025) and long terms (after 2025). Among the actions requested by the HLG-MR was a review of other potential methods for producing ^99^Mo in reactors and accelerators.

**Figure 3.**
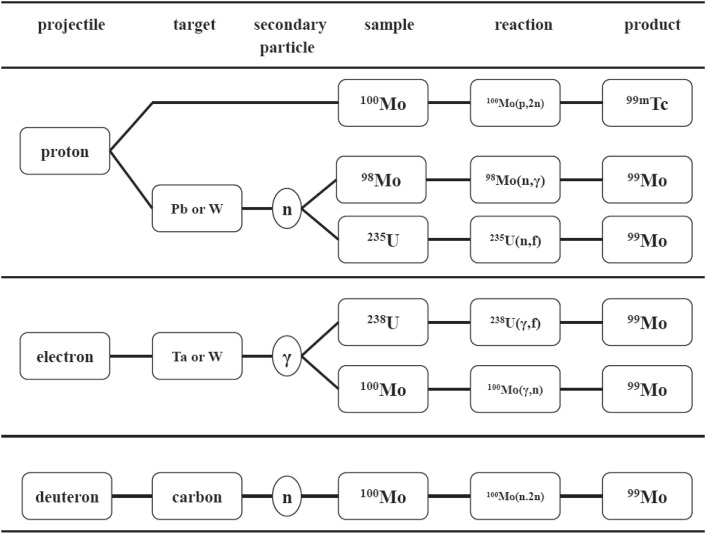
Typical examples of alternative production methods of ^99^Mo and ^99m^Tc. OECD. Nuclear Development (2010) The Supply of Medical Radioisotopes: Review of Potential Molybdenum-99/Technetium-99m Production Technologies. OECD. p. 17 (Ref. 23).

The OECD efforts have succeeded to significantly improve the global demand for ^99^Mo so as to meet at a near-to-full-service level. However, the fragility of the current production chain of ^99^Mo has been remaining, as mentioned above.^18)^ This vulnerable situation is an impetus to study alternative methods for producing ^99^Mo and/or ^99m^Tc in reactors or in accelerators worldwide. In fact, many efforts are being made for the production of ^99^Mo or ^99m^Tc worldwide. Typical proposed reactions are the (*n*,γ) reaction on ^98^Mo or natural Mo samples in reactors and the ^100^Mo(*p*,*pn*)^99^Mo, ^100^Mo(*d*,*p*2*n*)^99^Mo, ^100^Mo(*p*,2*n*)^99m^Tc, ^235^U(*n*,fission)^99^Mo, ^98^Mo(*n*,γ)^99^Mo, ^238^U(γ,fission)^99^Mo and ^100^Mo(γ,*n*)^99^Mo reactions in accelerators.^22)^ Reaction routes reported in the OECD report are shown in Fig. 3.^23)^

## 3 New production routes of medical radioisotopes using accelerator neutrons

### 3.1 ^99^Mo production via the ^100^Mo(*n*,*2n*)^99^Mo reaction.

The current route of fission-^99^Mo provides a large amount of ^99^Mo with a high specific activity of ∼370 TBq/(g ^99^Mo) in a single research reactor. Hence, a high specific activity of ^99m^Tc used for formulating ^99m^Tc radiopharmaceuticals is obtained from ^99^Mo using a commercially available ^99^Mo/^99m^Tc generator. Such highly specific activity of fission-^99^Mo is provided by high neutron flux of reactors, a large target volume of enriched ^235^U, and the high probability of the nuclear fission reaction of ^235^U. However, a specific activity of ^99^Mo based on any alternative routes, other than that of fission-^99^Mo, is as low as about 1/5,000 of the fission-^99^Mo. In fact, all alternative ^99^Mo production routes would face the challenges of lower reaction rates and lower specific activity, which must be overcome by fundamental technical breakthroughs. So far, a variety of nuclear reactions have been proposed to produce ^99^Mo in accelerators, as discussed in chapter 2. However, a production scheme of medical radioisotopes with fast neutrons from an accelerator (hereafter accelerator neutrons) without using a fissionable element U sample had not yet been considered.

In 2009, we proposed a new route to produce ^99^Mo by the ^100^Mo(*n*,2*n*)^99^Mo reaction using accelerator neutrons provided from an accelerator.^24)^ In proposing a scheme of ^99^Mo production using this route, we considered the following points. Firstly, any scheme of ^99^Mo production is required to meet all or a significant part of the domestic demand of ^99^Mo from an economic view point. We have kept in mind that because the requirement might be hardly met using existing accelerators, one has to propose the production scheme by taking into account the cost for an infrastructure, including an accelerator. Secondly, the safety and efficacy of the ^99m^Tc radiopharmaceutical preparation based on the proposed scheme should be ensured. Thirdly, we also took into account a criterion concerning the potential for other isotopes co-production at the same time, which was introduced in the OECD report.^23)^ It is considered to provide an indication of the economic sustainability, demand risk mitigation and the ability to avoid creating some other isotope shortage.

#### 3.1.1 Experiments and evaluations of the ^100^Mo(*n*,*2n*)^99^Mo reaction product.

**Figure 4.**
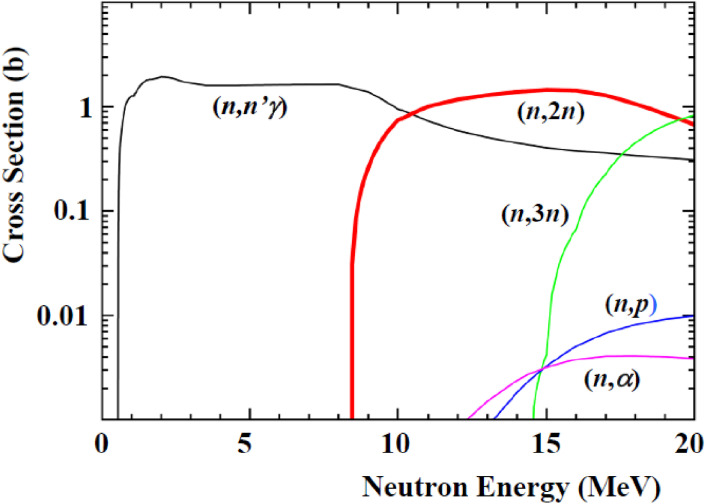
(Color online) Neutron-induced reaction cross sections on ^100^Mo.^26)^ J. Phys. Soc. Jpn. **82**, 064201 (Ref. 46).

In order to meet these requirements, the activity of ^99^Mo produced by a single accelerator should be as high as possible by considering the key parameters, such as the nuclear reaction cross section, beam flux, energy, irradiation time, number of sample nuclei, and half-life of the radioisotope, as discussed next. Firstly, the cross section of the ^100^Mo(*n*,2*n*)^99^Mo reaction has been measured^25)^ and evaluated^26)^ by many groups at a neutron energy of around 8 MeV up to 20.5 MeV. The ^100^Mo(*n*,2*n*)^99^Mo reaction cross section is the largest one (except for an elastic scattering cross section) in the neutron-induced reaction of ^100^Mo, about 1.5 barn in the neutron energy (*E*_*n*_) between 12 and 20 MeV, as shown in Fig. 4.^26)^ The cross sections of the ^100^Mo(*n*,α), ^100^Mo(*n*,3*n*), and ^100^Mo(*n*,*p*) reactions for producing impurity radioisotopes are less than a few mb at *E*_*n*_ ∼ 14 MeV. Hence, ^99^Mo could be produced with a minimum level of radioactive waste. Secondly, high-flux accelerator neutrons are expected to be provided by a neutron source based on the deuteron breakup reaction of light elements, such as carbon and beryllium *etc.* In fact, owing to the progress in accelerator technology, neutrons with a high flux of 10^15^ n/s having a most probable energy of 14 MeV are produced by the ^nat^C(*d*,*n*) reaction using 40 MeV, 5 mA deuterons at SPIRAL2 in GANIL in France.^27)^ The fluxes are compared with the thermal neutron flux of the reactor at Oak Ridge National Laboratory, having a factor × 10^15^ n/cm^2^/s.^28)^ The accelerator neutrons are characterized to be emitted in the forward-direction with respect to the deuteron beam direction. Hence, most of the emitted neutrons will be used effectively to produce ^99^Mo by placing a sample after a neutron target (discussed later) in the direction of the deuteron beam. Thirdly, a quantity of ^100^Mo samples of over 100 g weight mass can be used, because the neutron has no charge, and therefore the traveling range in a sample is much longer than that of a charged particle. When one uses a proton beam to produce ^99^Mo or ^99m^Tc via the ^100^Mo(*p*,*pn*)^99^Mo reaction or the ^100^Mo(*p*,2*n*)^99m^Tc reaction, the quantity of the ^100^Mo sample mass would be less than 1 g, owing to the short proton range in the ^100^Mo sample. In addition, proton beams have a heat problem of the sample because protons are stopped inside a ^100^Mo sample material, and therefore high-intensity proton beams of above a few hundred µA are hardly used to produce medical radioisotopes.

#### 3.1.2 ^99^Mo yield.

**Figure 5.**
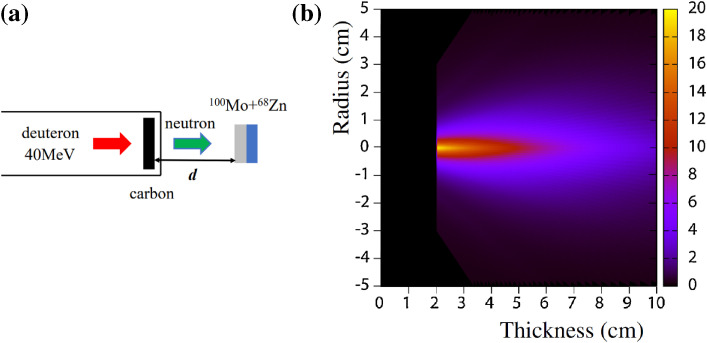
(Color online) (a) Schematic view of the setup of the carbon target and ^100^Mo sample. J. Phys. Soc. Jpn. **82**, 064201 (Ref. 46). (b) Calculated ^99^Mo yield distribution given in terms of the radius and thickness of a ^100^Mo sample. The color label of the right vertical axis indicates the yield of ^99^Mo (arbitrary unit). J. Phys. Soc. Jpn. **79**, 093201 (Ref. 29).

**Figure 6.**
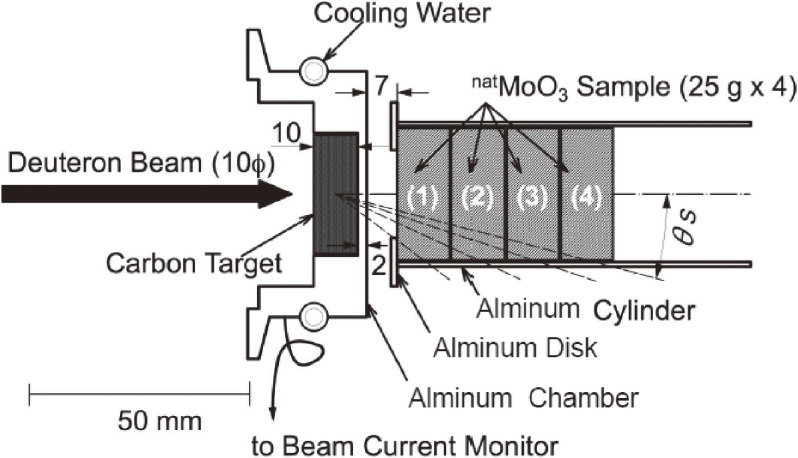
Schematic view of the experimental setup at the ^nat^MoO_3_ sample position. Sample numbers are indicated in parentheses. J. Phys. Soc. Jpn. **87**, 043201 (Ref. 32).

In obtaining the specific activity of the produced ^99^Mo as high as possible we calculated the angular and thickness distributions of the yield of ^99^Mo produced by irradiating a ^100^Mo sample having a large surface area with accelerator neutrons provided by the ^nat^C(*d*,*n*) reaction at a deuteron energy (*E*_d_) of 40 MeV.^29)^ In the calculation we assumed that the ^100^Mo sample would be placed 2 cm downward from the carbon target, as shown in Fig. 5a, and the 40 MeV deuteron beam size to be a point. We used the latest data of neutrons from the ^nat^C(*d*,*n*) reaction at *E*_d_ = 40 MeV^30)^ and the evaluated cross section of ^100^Mo(*n*,2*n*)^99^Mo given in the Japanese Evaluated Nuclear Data Library (JENDL-4.0).^26)^ As we can see in Fig. 5b, the calculated ^99^Mo yield is mostly distributed in a narrow region at an extremely forward angle with respect to the deuteron beam direction and within a sample thickness of as thick as about 4 cm. From this result, an appropriate shape of the ^100^Mo sample to obtain a high specific activity of ^99^Mo is determined to be cylindrical. Next, we measured the yield of ^99^Mo produced by the ^nat^Mo(*n*,2*n*)^99^Mo reaction to make a rigorous test of the measured energy and angular distributions of the accelerator neutrons, including the evaluated cross section.^31,32)^ We used four pellet ^nat^MoO_3_ samples of 25.869, 25.868, 25.483 and 25.220 g mass (in total 102.440 g mass) with dimensions of 30 mmϕ × 11.6 mm (total length 46.4 mm), as shown in Fig. 6.^32)^ The isotopic composition of ^nat^Mo is 14.8% for ^92^Mo, 9.25% for ^94^Mo, 15.92% for ^95^Mo, 16.68% for ^96^Mo, 9.55% for ^97^Mo, 24.13% for ^98^Mo and 9.63% for ^100^Mo.^33)^ The accelerator neutrons were provided by the ^nat^C(*d*,*n*) reaction using 40 MeV deuterons at the azimuthally variable field (AVF) cyclotron at Cyclotron and Radioisotope Center (CYRIC), Tohoku University.^34)^ The distance, *d*, between the carbon target and the ^nat^MoO_3_ sample was 9 mm.

The activity of ^99^Mo at the end of irradiation (EOI) was determined, as given in Table 4, by considering the branching ratio of the observed γ-rays and the γ-ray detection efficiency of the HPGe detector. The self-absorption of the γ-rays in the irradiated ^100^MoO_3_ sample was corrected by using a photon cross-sectional database provided by the National Institute of Standards and Technology.^35)^ Next, we estimated the yield of ^99^Mo by numerical calculations for a comparison with the measured yield, as follows. We used the neutron-nucleus reaction cross sections given in the fourth version of the Japanese Evaluated Nuclear Data Library (JENDL-4.0) for molybdenum and oxygen in the ^100^MoO_3_ sample. In calculating the neutron flux we used the latest data of the cross section, which was obtained by irradiating a 15-mm-thick carbon target with 40 MeV deuteron beams.^30)^ We corrected for the difference in the attenuation of the neutron flux inside the carbon target because the thickness of the carbon target used in the aforementioned study^30)^ was 15 mm, compared with 10 mm in this measurement. The correction was made by using the simulation code Particle and Heavy Ion Transport code System (PHITS).^36)^

The estimated yield of ^99^Mo for each set of the ^nat^Mo samples is in good agreement with the measured yield, as given in Table 4, which reflects the accuracies of the measured neutron energy and the angular distributions of the neutrons from the ^nat^C(*d*,*n*) reaction and the accuracy of the evaluated cross section of the ^100^Mo(*n*,2*n*)^99^Mo reaction.

Based on the good agreement between the measured ^99^Mo yield and the calculated yield, we calculated the activities of ^99^Mo at the EOI, produced by the ^100^Mo(*n*,2*n*)^99^Mo reaction for an enriched ^100^MoO_3_ sample, in terms of the weight and radius (*r*_s_) of the ^100^MoO_3_ sample, the distance (*d*) between the carbon target and the ^100^MoO_3_ sample, and the irradiation time (*i*_t_).^31)^ In the calculation, we used the latest data on the angular and energy distributions of neutrons from ^nat^C(*d*,*n*) at *E*_d_ = 40 MeV (assuming a beam intensity of 2 mA) and evaluated cross sections given in JENDL-4.0.^26)^ Some of the calculated ^99^Mo yields are listed in Table 5. Here, we took the radius of the deuteron beam, *r*_d_, of 0.5 cm and *i*_t_ = 24 h as a typical setup to reduce the heat power density in the carbon target deposited by the deuteron beam and the decay loss of ^99^Mo produced during the neutron irradiation period. We can see that both the ^99^Mo and ^99m^Tc yields have a maximum at *r*_s_ = 2.0 cm independent of *d* in most cases.

#### 3.1.3 Capability to meet demand for ^99^Mo/^99m^Tc in Japan.

In order to calculate the activity of ^99m^Tc, which is obtained daily using ^99^Mo produced every day by the ^100^Mo(*n*,2*n*)^99^Mo reaction, we first calculated the ^99^Mo activity reserved daily. Secondly, we compared the calculated activity with the current demand of ^99^Mo, which is estimated by considering the number of diagnostic procedures by using ^99m^Tc-radiopharmaceuticals every year in Japan.^31)^

**Figure 7.**
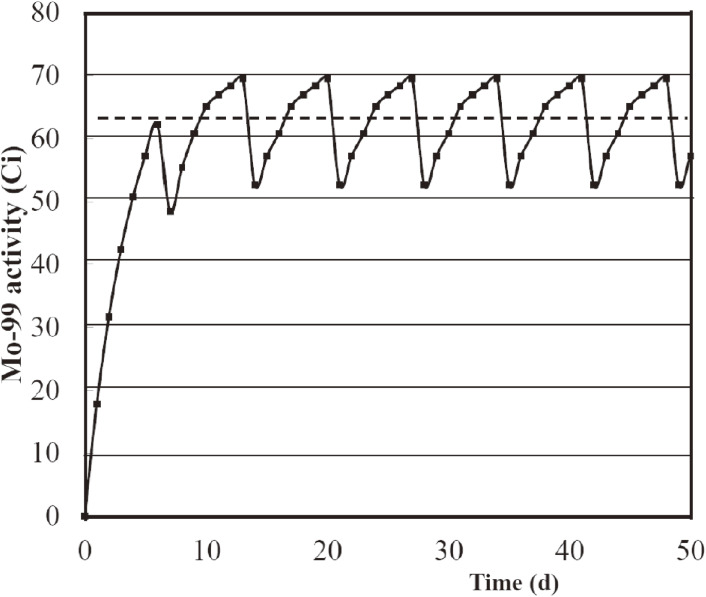
Calculated ^99^Mo activity after starting irradiation of the ^100^MoO_3_ sample with neutrons as a function of time (day). When 657 GBq (18 Ci) of ^99^Mo is produced daily for 6 days per week, 2.30 TBq (63 Ci) of ^99^Mo (in total) is obtained daily on average (dotted line). J. Phys. Soc. Jpn. **86**, 114803 (Ref. 31).

As given in Table 5 we calculated that a certain amount of ^99^Mo is produced every day by the ^100^Mo(*n*,2*n*)^99^Mo reaction with 40 MeV, 2 mA deuteron beams (24 h irradiation). Through our studies discussed later we consider that an enriched ^100^MoO_3_ sample of mass 150 g (100 g of ^100^Mo) is one of the feasible cases in terms of the present production efficiency of ^99^Mo, and the elution performance of ^99m^Tc from ^99^Mo, and the shortest distance between the carbon target and the ^100^MoO_3_ sample will be practically *d* = 1.0 cm by taking account of the mechanical structure of a rotating carbon target system (discussed later). Since we obtain the maximum (calculated) yield of ^99^Mo for a sample radius of *r* = 2.0 cm for most cases (Table 5), the calculated yield of ^99^Mo at the EOI per day is 657 GBq (150 g ^100^MoO_3_ sample mass, *r*_s_ = 2.0 cm, and *d* = 1.0 cm). The daily production of 657 GBq (18 Ci) of ^99^Mo provides 2.30 TBq (63 Ci) of ^99^Mo on average after 10 days in steady-state operation of the cyclotron, as shown in Fig. 7. It should be mentioned that we can also obtain the ^99^Mo yield of 700 GBq using a ^100^MoO_3_ sample of 100 g by employing a following neutron irradiation scheme of the sample. The calculated yields of ^99^Mo for one-pellet (100 g) and two-pellet (2 × 100 g) ^100^MoO_3_ samples are 505 and 756 GBq (Table 5), respectively. Hence, the yield of ^99^Mo for one of the two samples, which was placed on the far side of the carbon target [hereafter the second sample] during the first irradiation time of 24 h, is 251 GBq. During the second neutron irradiation time of 24 h, the second sample was placed on the near side of the carbon target, resulting in a ^99^Mo yield of 505 + 251 × 0.78 = 700 GBq. This yield is approximately the same (657 GBq) as that using a 150 g ^100^MoO_3_ sample mass. The factor 0.78 is due to the decay of ^99^Mo, which was produced in the first irradiation. Hence, a 100 g ^100^MoO_3_ sample mass would be a better choice from an economical view point.^32)^

Hence, by eluting ^99m^Tc from the reserved ^99^Mo of 2.30 TBq once a day we obtain on average 1.63 TBq (44 Ci) of ^99m^Tc daily. Note that the activity of ^99m^Tc at time *t*, (*A*_Tc_)_*t*_, is calculated by using the following equation^37)^:

$$(A_{\text{Tc}}{)}_{t}=\frac{0.875\lambda_{2}}{\left(\lambda_{2}-\lambda_{1}\right)}(A_{\text{Mo}}{)}_{0}(e^{-\lambda_{1}t}-e^{-\lambda_{2}t}),$$ [1]

where λ_1_ = 0.0105/h and λ_2_ = 0.1155/h are the decay constants for ^99^Mo and ^99m^Tc, respectively, and (*A*_Mo_)_0_ is the activity of ^99^Mo at *t* = 0.

**Figure 8.**
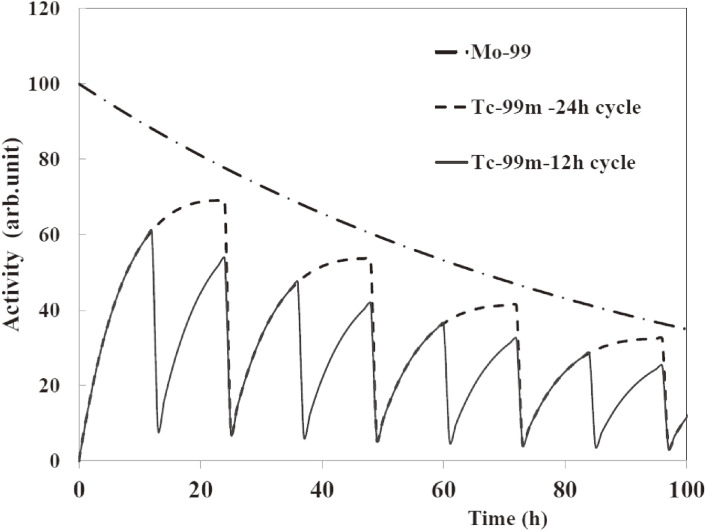
Decay-growth curve of the ^99^Mo and ^99m^Tc. ^99m^Tc is eluted either every 12 h or every 24 h. J. Phys. Soc. Jpn. **86**, 114803 (Ref. 31).

Next, the ^99m^Tc activity that might be obtained daily at the end of thermochromatographic separation, discussed later, is evaluated. After the EOI, the irradiated ^100^MoO_3_ sample will be placed in a thermochromatography apparatus to separate ^99m^Tc from the neutron irradiated sample containing ^99^Mo. Taking into account the decay loss of ^99m^Tc during the separation procedure for about 2 h and the separation efficiency of 80%, the ^99m^Tc activity immediately after separating ^99m^Tc from the “old” and “new” ^99^Mo is 1.30 TBq (35 Ci). Here, “old” and “new” ^99^Mo indicate the activity of ^99^Mo produced on previous days and that day, respectively. Note that a ^99m^Tc solution is usually eluted twice per day, and therefore the obtained ^99m^Tc activity is 2.21 TBq (60 Ci), 1.7-times that in the case of elution once a day (see Fig. 8).

We next discuss the demand of ^99^Mo in Japan by considering the number of diagnostic procedures currently carried out by using ^99m^Tc radiopharmaceuticals. About 0.9 million diagnostic procedures (2,750 procedures/day) have been performed every year using ^99m^Tc radiopharmaceuticals with an average dose of about 740 MBq (20 mCi) at the time of injection to a patient: 2.05 TBq (55 Ci) of ^99m^Tc is used every day. The activity of ^99m^Tc needed in Japan every day at a radiopharmaceutical company to prepare ^99m^Tc radiopharmaceuticals is calculated to be 4.10 TBq (110 Ci) by referring to the reports by Pillai *et al.*,^38)^ Bennett *et al.*,^39)^ and Ross *et al.*^40)^ as follows. In the U.S.A., about 50,000 diagnostic procedures are carried out daily using ^99m^Tc radiopharmaceuticals. The U.S.A. requires 55.5 TBq (1,500 Ci) of ^99m^Tc daily assuming that 1.1 GBq (30 mCi) of ^99m^Tc is injected into a patient, and by considering the decay loss during the transportation of ^99m^Tc from the radiopharmaceutical company to hospitals, a total ^99m^Tc activity of 111 TBq (3,000 Ci) is required every day. Similarly, the total ^99m^Tc activity required in Japan is calculated to be 4.10 TBq (110 Ci) every day.

As discussed above, 2.21 TBq (60 Ci) of ^99m^Tc is obtained at the end of the separation of ^99m^Tc, *i.e.*, immediately before forming the ^99m^Tc radiopharmaceuticals at a radiopharmaceutical company. When one can inject the ^99m^Tc radiopharmaceuticals prepared by using the 2.21 TBq ^99m^Tc within 6 h to a patient, about 50% of the daily procedures using ^99m^Tc radiopharmaceuticals can be performed in Japan. In fact, because about 35% of Japan’s population live in the capital-area (a region within about 150 km radius from the center of Tokyo, *e.g.*, Kanagawa, Saitama, Chiba, Ibaraki, Tochigi, Gunma, and Yamanashi prefectures), and the number of people who live in the capital-area, Kansai-area, and Chukyo-area is about 60% of the total population of Japan, ^99m^Tc radiopharmaceuticals can be delivered from a radiopharmaceutical company to those who live there within 6 h. The proposed delivery of ^99m^Tc would be possible by using the current delivery system of ^18^F-FDG,^41)^ a radiopharmaceutical fluorodeoxyglucose containing ^18^F with a half-life of 1.8 h shorter than ^99m^Tc (*T*_1/2_ = 6 h). In Japan, deliveries of ^18^F-FDG are being carried out three times per day by road transport from the ^18^F-FDG-producing radiopharmaceutical company to hospitals about 200 km away within 3 h. The activity of ^18^F prepared at the radiopharmaceutical company is three-times stronger than that needed for injection into a patient at a hospital by considering a decay loss of 68% during the transport of ^18^F in 3 h. Note that the ^99m^Tc activity needed at hospitals for the ^99m^Tc procedures to satisfy 50% of the demand in Japan is 10.2 TBq (27.5 Ci). The decay loss of ^99m^Tc activity in transportation for 3 h is 30%. Hence, the ^99m^Tc activity of 60 Ci obtained at a radiopharmaceutical company would be enough to perform the mentioned procedures. Note that we decided to harvest the produced ^99^Mo every day by considering the ^100^Mo inventory and the ^99^Mo decay during the ^100^Mo sample irradiation. A harvest frequency of ^99^Mo once every six days would decrease the instantaneous production rate by about 40% relative to that of one day.

#### 3.1.4 Radionuclide purity of ^99^Mo.

The radionuclide purity of ^99^Mo should be high in order to obtain high-quality ^99m^Tc to perform the separation process of ^99m^Tc from ^99^Mo under radionuclides with little impurities, and not to create a problem for the storage of long-lived radioactivity. We measured the radionuclide purity of ^99^Mo produced by using an enriched ^100^MoO_3_ sample of 0.578 g. The isotopic composition of Mo in the enriched ^100^MoO_3_ sample was 0.15 at% for ^94^Mo, 0.1 at% for ^94^Mo, 0.18 at% for ^95^Mo, 0.21 at% for ^96^Mo, 0.17 at% for ^97^Mo, 3.29 at% for ^98^Mo, and 95.90 at% for ^100^Mo. The neutrons were provided by the ^nat^C(*d*,*n*) reaction using 40 MeV, 1.75 µA deuterons at the Takasaki Ion Accelerators for Advanced Radiation Application of the National Institutes for Quantum and Radiological Science and Technology (hereafter TIARA-QST).^42)^ The ^100^MoO_3_ sample was irradiated for 5 h.

**Figure 9.**
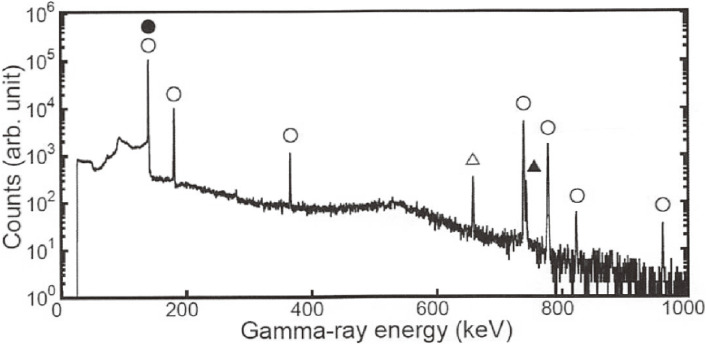
γ-ray spectrum of the ^100^MoO_3_ sample taken 4 h after the EOI. The γ-ray peaks are from the decay of ^99^Mo (open circles), ^99m^Tc (filled circle), ^97^Zr (open triangle), and ^97^Nb (filled triangle). J. Phys. Soc. Jpn. **86**, 114803 (Ref. 31).

A typical γ-ray spectrum of the ^100^MoO_3_ sample irradiated by neutrons is shown in Fig. 9. We clearly observed only several γ-rays from the decay of ^99^Mo (at 181.1 keV) and ^99m^Tc (at 140.5 keV), and impurity γ-rays from ^97^Zr (*T*_1/2_ = 16.9 h at 743.4 keV) and ^97g^Nb (*T*_1/2_ = 1.2 h at 658.1 keV).

The activities of ^99^Mo and ^97^Zr at the EOI were determined to be (3.16 ± 0.12) × 10^6^ Bq for ^99^Mo and (31.5 ± 1.6) × 10^3^ Bq for ^97^Zr, which is 1% of the ^99^Mo activity, as shown in Table 6. Namely, ^99^Mo was produced with a minimum level of radioactive waste and without radioisotopes of Tc other than ^99m^Tc and ^99^Tc (*T*_1/2_ = 2.1 × 10^5^ y). They are important because the irradiated enriched ^100^MoO_3_ sample can be recycled.

### 3.2 Therapeutic radioisotopes production by the (*n*,*xp*) or (*n*,*αx*) reaction.

#### 3.2.1 Medical radioisotopes produced in reactors and accelerators.

**Figure 10.**
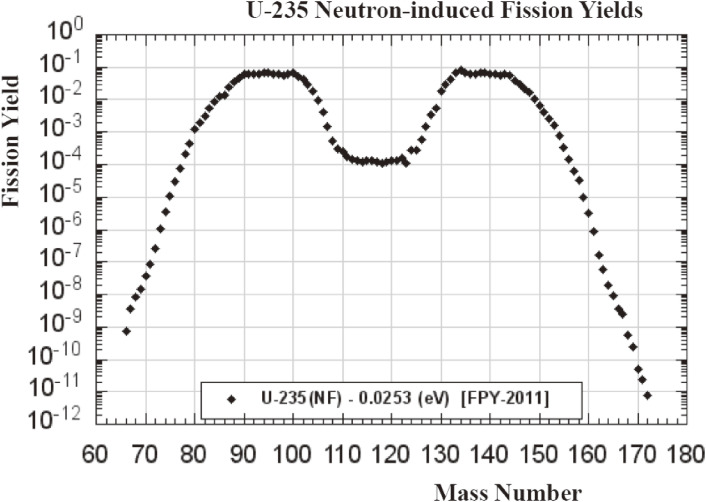
^235^U Fission product mass yield curve. No. JAEA-DATA/CODE--2011-025. Tokai, Japan: Japan Atomic Energy Agency, 2012 (Ref. 43).

In the treatment for patients with cancers, medical RIs are used first to obtain pre-therapy imaging information concerning biodistribution and dosimetry in patients, and second to perform higher dose targeted molecular therapy in the same patients. Most medical RIs used for imaging and therapy are, respectively, being produced in accelerators (except ^99^Mo) and in reactors (except ^225^Ac). In the production of therapeutic RIs in reactors by the fission reaction of ^235^U or the thermal neutron capture reaction of a sample, high thermal neutron fluxes on the order of 10^14^ n/(cm^2^ s) and the use of a large quantity of a sample plays a key role. Note that carrier-free (without any isotope of a sample nuclide of ^235^U) RIs suitable for medical use are obtained by the fission reaction. Currently, carrier-free RIs of ^90^Y (*T*_1/2_ = 2.67 d), the daughter radioisotope of ^90^Sr (*T*_1/2_ = 28.8 y), and ^131^I (*T*_1/2_ = 8.02 d) have been used for therapy. On the other hand, carrier-added (with a sample) RIs are usually generated by the thermal-neutron capture reaction. They cannot be separated from a neutron-irradiated sample because the atomic number of RIs is the same as that of a sample. However, there are several cases in which the produced RIs can decay by emitting β^−^-rays, resulting to obtain carrier-free RIs by chemical separation. ^131^I and ^177^Lu are, respectively, produced by the ^130^Te(n,γ)^131^Te → ^131^I and ^176^Yb(n,γ)^177^Yb → ^177^Lu reactions. Here, it must be noted that currently constantly available RIs produced by the fission reaction and thermal neutron capture reaction are, ^89^Sr, ^90^Y, ^131^I, ^177^Lu, ^192^Ir, and ^198^Au *etc.* We might expect that a wide variety of medical radioisotopes can be produced in reactors by the two reactions mentioned above. A limitation of available numbers comes mainly from the fission yield curve of ^235^U having maxima at masses of around 90–100 and 133–143, as shown in Fig. 10, and a sample mass dependence of the thermal neutron capture reaction having a large cross section.^43)^

In accelerators a wide variety of carrier-free RI with a high specific activity, mostly used for diagnostics, have been produced by using proton beams. In proton irradiation on a sample, it must be noted that the whole proton energy is transformed into heat in the sample, and the traveling range of protons in a sample is much shorter than that of neutrons, which limit both the proton beam intensity and the quantity of the sample for producing RIs. Hence, therapeutic RIs are hardly produced using proton beams. We first proposed a new route to produce ^99^Mo by using accelerator neutrons, and then proposed new methods to produce therapeutic RIs.^44–46)^

#### 3.2.2 Production for theranostic radioisotopes.

**Figure 11.**
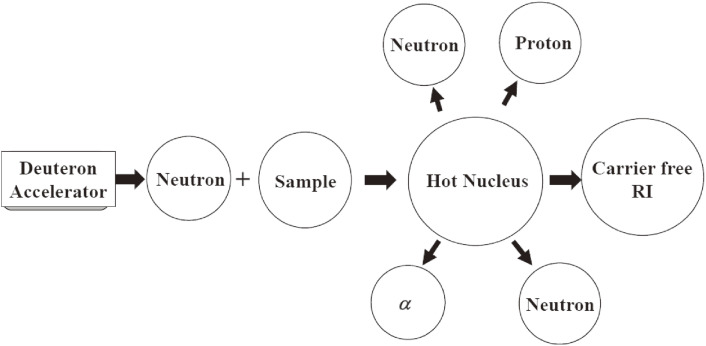
Schematic view of the production of a wide variety of therapeutic radioisotopes. A hot nucleus is produced by bombarding a sample with accelerator neutrons, followed proton, neutron or α-particle emission. When a charged particle, such as a proton or α, is emitted, one can obtain carrier-free RIs by employing a chemical separation technique.

A charge-exchange reaction, such as (*n*,*p*), (*n*,*x*), and (*n*,α), of a sample nucleus with a medium-weight mass, has a sizable cross section of from ∼50 to ∼500 mb at a neutron energy of between ∼10 and ∼30 MeV, which is almost independent of the mass number of the sample. Here, (*n*,*x*) denotes the (*n*,*n*′*p*) and (*n*,*d*) reactions. The cross section of the (*n*,2*n*) reaction of a neutron-rich nucleus at ∼10 < *E*_n_ < 20 MeV is also quite large, and does not depend so much on the nuclear mass; it is in the range between 500 and 2,000 mb. Note that the neutron has no charge, and therefore the traveling range in a sample is much longer than that of a charged particle. Therefore, using high flux accelerator neutrons and a large amount of a sample, a large quantity of a wide variety of carrier-added and carrier-free radioisotopes (without any other isotope of a sample nuclide) can be produced, which would lead to a new era in theranostic RIs production. A schematic view of the production of a variety of therapeutic radioisotopes using accelerators and many stable isotopes is shown in Fig. 11.

We have proposed new routes to produce carrier-free medical radioisotopes of ^90^Y,^43)^ ^64^Cu, and ^67^Cu^44,45)^ using accelerator neutrons provided by the ^nat^C(*d*,*n*) reaction. Successful PET with the use of ^18^F for assessments of tumor characterization has triggered a search for a longer half-life PET RI to diagnose the dynamics of a medicine in a living body that has a slow reaction time. ^64^Cu with a half-life of *T*_1/2_ = 12.7 h, longer than that of ^18^F (*T*_1/2_ = 1.8 h), is considered to be a promising RI suitable for labeling many radiopharmaceuticals for PET imaging,^2)^ since ^64^Cu decays by positron (β^+^) emission. The Cu radioisotope is known to have unique potentials useful for diagnostic imaging and in targeted radionuclide therapy.^4,5)^ In radioimmunotherapy (RIT) for tumor treatments, ^90^Y (*T*_1/2_ = 64 h), a pure β^−^-ray emitter with an average β^−^-ray energy of 935 keV, is most widely used to kill large tumor masses, since the range of β^−^-rays in H_2_O is as long as 12 mm. ^67^Cu (*T*_1/2_ = 62 h), a pair radioisotope of ^64^Cu, is considered to be a promising radionuclide for treating small distant metastases of up to 4 mm in size in radioimmunotherapy.^6)^ ^67^Cu has unique nuclear properties and chemical behavior for use in RIT.^4,5)^ Namely, ^67^Cu emits β^−^-rays with an average energy of 141 keV, which allow radiopharmaceuticals of ^67^Cu to provide a lethal dose of radiation to target cancer cells. ^67^Cu also emits 185 keV γ-rays, which permit SPECT imaging during therapy. In addition, ^67^Cu has a sufficiently long half-life (*T*_1/2_) of 62 h, allowing it to be delivered to tumors, which may take 24 to 48 h to reach their peak concentration in tumors.^4)^ ^64^Cu should be noted to be used for pre-therapeutic PET studies for accurate evaluations of the dose delivered to a normal organ before the injection of ^67^Cu for RIT to patients, since the irradiation of vital organs should be minimized. The coordination chemistry of copper applied to the production of radiopharmaceuticals has been well established. On the basis of a successful clinical study on a radiopharmaceutical containing ^67^Cu for B-cell non-Hodgkin lymphoma, about 450 TBq (12,000 Ci) of ^67^Cu is considered to be required per year in the U.S.A.^47)^ Currently, there exists no technology to meet such a demand.

Thus far, many studies have been carried out to produce ^64^Cu and ^67^Cu in reactors or accelerators. ^64^Cu was produced by the ^64^Zn(*n*,*p*)^64^Cu reaction in reactors using accelerators by the ^64^Ni(*p*,*n*)^64^Cu, ^64^Ni(*d*,2*n*)^64^Cu, ^64^Zn(*d*,2*p*)^64^Cu, ^66^Zn(*d*,α)^64^Cu, ^68^Zn(*p*,α*n*)^64^Cu, and ^64^Zn(*n*,*p*)^64^Cu reactions in accelerators.^47)^ Among the studies, the generally adopted production route is the ^64^Ni(*p*,*n*)^64^Cu reaction, which provides a high specific activity ^64^Cu using a highly enriched ^64^Ni target. The maximum production yield of ^64^Cu is expected to be about 37 GBq (1 Ci) by bombarding a ^64^Ni sample with 50 µA proton beams for 12 h.^48)^ It is very greatly encouraged to increase the availability of ^64^Cu. ^67^Cu has been produced by the ^67^Zn(*n*,*p*)^67^Cu reaction in both reactors and accelerators,^13)^ and in accelerators via the ^68^Zn(*p*,2*p*)^67^Cu, ^70^Zn(*p*,α)^67^Cu, ^64^Ni(α,*p*)^67^Cu, ^68^Zn(γ,*p*)^67^Cu and ^67^Zn(*n*,*p*)^67^Cu reactions. Note that the isotopic components of natural Zn are 48.6% ^64^Zn, 27.9% ^66^Zn, 4.1% ^67^Zn, 18.8% ^68^Zn, and 0.62% ^70^Zn.^33)^ Among the reactions, the ^68^Zn(*p*,2*p*)^67^Cu and ^68^Zn(γ,*p*)^67^Cu reactions are currently used for the production of ^67^Cu. Since the proton energy used in the ^68^Zn(*p*,2*p*)^67^Cu reaction is high, a large amount of impurity RI of ^64^Cu is produced by the ^68^Zn(*p*,α*n*)^64^Cu reaction at EOI. Regarding the ^67^Cu production induced by accelerator neutrons, the spectrum-averaged cross section of the ^67^Zn(*n*,*p*)^67^Cu reaction was measured at *E*_n_ = 4.95 MeV,^49)^ but the route has not yet been adopted because of the small cross section and the lack of an intense neutron source. Note that recently high-quality ^67^Cu has been produced by the ^68^Zn(γ,p)^67^Cu reaction at Argonne National Laboratory.^50)^

Concerning the ^64^Cu and ^67^Cu productions using accelerator neutrons, the main drawback comes from the low neutron flux, but not from the nuclear reaction processes, such as the cross section of a required RI or the high production yield of an impurity RI. As mentioned above a high neutron flux of ∼10^15^ n/s at an average neutron energy of *E*_n_ ≈ 14 MeV can be obtained owing to recent progress in accelerator technology. These findings led us to propose new routes to produce carrier-free radioisotopes of ^64^Cu by the ^64^Zn(*n*,*p*)^64^Cu reaction and ^67^Cu via the ^67^Zn(*n*,*p*)^67^Cu and ^68^Zn(*n*,*x*)^67^Cu reactions using accelerator neutrons. In order to calculate the production yields of ^64^Cu and ^67^Cu we first re-measured the cross sections of the ^64^Zn(*n*,*p*)^64^Cu, ^67^Zn(*n*,*p*)^67^Cu, and ^68^Zn(*n*,*x*)^67^Cu reactions using neutrons at *E*_n_ ≈ 14 MeV. Although many studies were carried out to measure those cross sections at *E*_n_ ≈ 14 MeV, there remained significant differences between different data sets. The measurement was carried out using ∼14 MeV neutrons produced via the ^3^H(*d*,*n*)^4^He reaction at the Fusion Neutronics Source (FNS) facility of Japan Atomic Energy Agency (JAEA).^51)^ The obtained results led us to estimate the yields of ^64^Cu produced by the ^64^Zn(*n*,*p*)^64^Cu reaction and ^67^Cu by the ^67^Zn(*n*,*p*)^67^Cu and ^68^Zn(*n*,*x*)^67^Cu reactions using high neutron fluxes. The neutrons could be provided by the ^nat^C(*d*,*n*) reaction with 40 MeV, 5 mA deuteron beams.^27)^ The estimation was performed using the evaluated cross section of the neutron induced reaction on Zn isotopes given in the Japanese Evaluated Nuclear Data Library. The yield of ^64^Cu was calculated to be 1.8 TBq/(175 g of 100%-enriched sample of ^64^Zn) for an irradiation time of 12 h, which is much larger than the expected ^64^Cu yield of 37 GBq via ^64^Ni(*p*,*n*)^64^Cu, and that of ^67^Cu via the ^68^Zn(*n*,*x*)^67^Cu reaction was calculated to be 287 GBq/(186 g of 100%-enriched sample of ^68^Zn) at EOI for an irradiation time of 2 days,^45)^ which is much larger than a reported yield of 10 GBq by ^nat^Zn(*p*,2*p*)^67^Cu at *E*_*p*_ = 192 MeV at an average beam current of 43 µA for a period of 5–6 days.^52)^

#### 3.2.3 Radionuclide purity of ^67^Cu.

**Figure 12.**
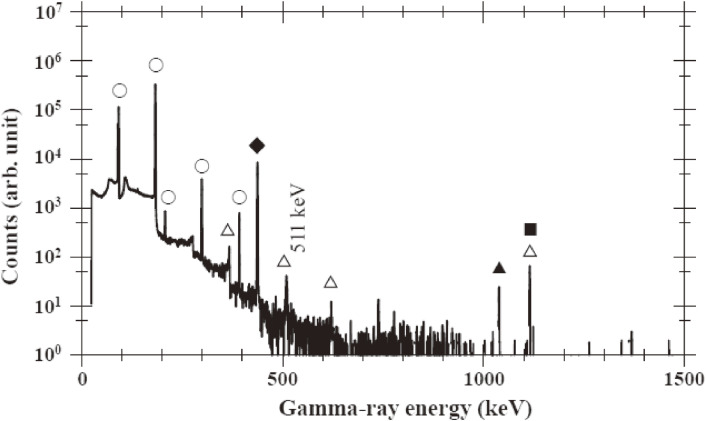
γ-ray spectrum of a ^68^ZnO sample irradiated with neutrons. The γ-ray peaks come from the decays of ^67^Cu (open circles), ^65^Ni (open triangles), ^66^Ni (filled triangle), and ^69m^Zn (filled diamond), and ^65^Zn (filled square). The spectrum was taken 40 h after EOI. J. Phys. Soc. Jpn. **83**, 073201 (Ref. 53).

We have studied the radionuclide purity of ^67^Cu,^53)^ the vital importance for its medical use and the recycling of an enriched sample of ^68^Zn, produced by bombarding an enriched ^68^ZnO sample of 0.329 g for 5 h with neutrons from the ^nat^C(*d*,*n*) reaction at *E*_d_ = 40 MeV at an average beam current of 1.84 µA at TIARA-QST. The isotopic compositions of Zn in the enriched ^68^ZnO sample were 0.047 at% for ^64^Zn, 0.104 at% for ^66^Zn, 0.466 at% for ^67^Zn, 99.291 at% for ^68^Zn, and 0.092 at% for ^70^Zn. A typical background-subtracted γ-ray spectrum of the irradiated ^68^ZnO sample is shown in Fig. 12. We observed γ-rays from the decay of ^67^Cu (at 91, 93, 185, 209, 300, and 394 keV), ^65^Ni (*T*_1/2_ = 2.52 h at 366, 508, 610, and 1,116 keV), ^66^Ni (*T*_1/2_ = 54.6 h at 1,039 keV), ^65^Zn (*T*_1/2_ = 244 d at 1,116 keV), and ^69m^Zn (*T*_1/2_ = 13.8 h at 439 keV). The isotope assignments of the γ-rays were made on the basis of their energies and decay curves. ^65^Zn was identified by the 1,116 keV γ-ray.

The radionuclide purity of ^67^Cu was determined to be extremely low compared with those produced by the ^68^Zn(*p*,2*p*)^67^Cu and ^70^Zn(*d*,α*n*)^67^Cu reactions, as given in Table 7, and estimated ones to investigate a possible reaction for producing impurity radionuclides. The estimation was made using the isotope composition of the enriched ^68^ZnO sample mentioned above, the neutron energy spectra from the ^nat^C(*d*,*n*) reaction, and neutron nuclear reaction cross-sectional data on Zn isotopes.^26)^ The estimated activity ratios of the impurity radioisotopes agree with the experimental ratios within the experimental uncertainties, as given in Table 7.^53)^ The obtained information is also important when purchasing an expensive sample with a variety of isotopic compositions.

### 3.3 Production of radioisotopes in polyethylene blocks.

We have proposed another production route by using accelerator neutrons backscattered by materials, such as polyethylene or lead blocks.

#### 3.3.1 Experiment for producing RIs using polyethylene blocks.

In studying the production routes of medical RIs with accelerator neutrons by using a sample that was covered with polyethylene blocks to reduce the neutron background in an experimental room, we happened to find much larger yields of some of the RIs than those without polyethylene blocks.^54)^

**Figure 13.**
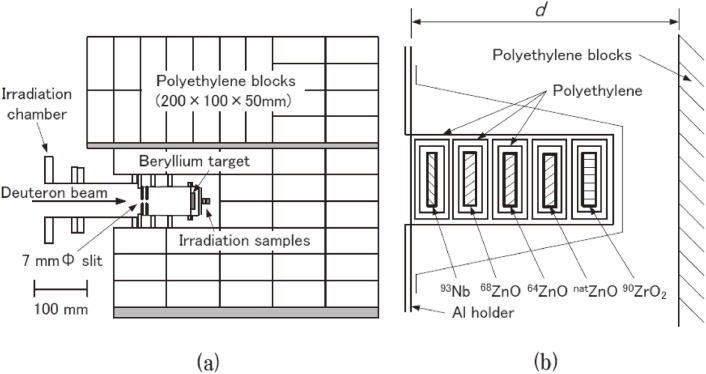
Schematic view of the experimental setup at the position of samples in polyethylene blocks (a), and the five stacked samples (b). *d* (cm) is the distance between the Al holder and the polyethylene block. J. Phys. Soc. Jpn. **89**, 034201 (Ref. 45).

This study was performed by irradiating five stacked samples of ^93^Nb, enriched ^68^ZnO, enriched ^64^ZnO, natural ^nat^ZnO, and enriched ^90^ZrO_2_ as well as two stacked samples of ^93^Nb and enriched metallic ^68^Zn with accelerator neutrons. The ^93^Nb sample was used as a high-energy neutron-fluence monitor.^54)^ The masses for the ^68^ZnO, ^68^Zn, and ^64^ZnO samples were about 360 mg, and the enrichment of the ^68^ZnO, ^68^Zn, and ^64^ZnO samples was over 99%. These samples were covered with polyethylene (or lead) blocks, as shown in Fig. 13a. The size of the individual polyethylene or lead block was 200 × 100 × 50 mm^3^. The distance (*d*) between the Al holder and the polyethylene (or lead) shown in Fig. 13b was set to be either 3 or 6 cm for investigating a possible effect of the blocks on the yields of produced RIs. The neutrons were provided from the deuteron breakup reaction on a ^9^Be target using a 50 MeV, 0.5 µA deuteron beam at TIARA-QST. The samples were irradiated for about 15 min.

**Figure 14.**
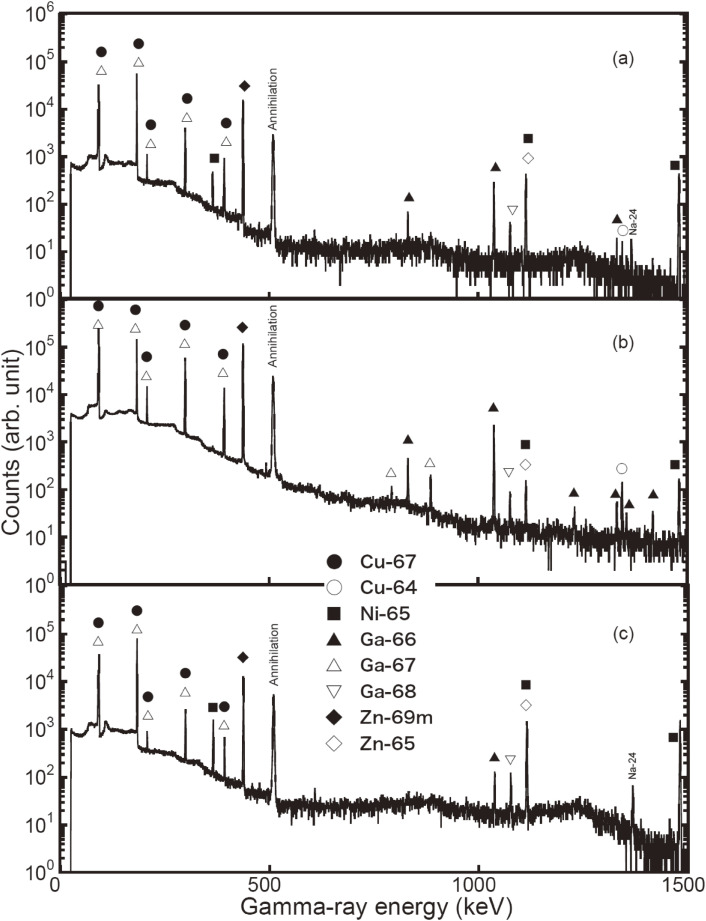
γ-ray spectra from the decay of produced radioisotopes that were taken about 15 min after the EOI for the ^68^ZnO sample (a) without polyethylene blocks and (b) with polyethylene blocks, and (c) for a metallic ^68^Zn sample with polyethylene blocks placed at *d* = 3 cm. J. Phys. Soc. Jpn. **89**, 034201 (Ref. 45).

Hereafter we focus on the results of the two samples, ^68^ZnO and ^68^Zn; ^68^Zn(PE) and ^68^Zn(Pb) indicate ^68^Zn samples covered with polyethylene and with lead, and ^68^Zn(no) stands for a ^68^Zn sample with neither polyethylene nor lead, respectively. Typical γ-ray spectra of the irradiated ^68^ZnO(no), ^68^ZnO(PE), and ^68^Zn(PE) samples placed at *d* = 3 cm are shown in Figs. 14a, 14b, and 14c, respectively. Identifications of the produced radioisotopes were made based on the γ-ray energies and/or the absolute γ-ray branching ratio (*I*_γ_), as given in Table 8.

An anomalous nuclear reaction phenomenon was found in this study. Namely, significant amounts of proton-induced reaction products of ^66^Ga and ^67^Ga were observed when irradiating an enriched ^68^ZnO(PE) sample with accelerator neutrons. In fact, the γ-ray peak intensities of ^66^Ga (at 834 and 1,039 keV), ^67^Ga (at 300 keV), ^69m^Zn (at 439 keV), and ^64^Cu (at 1,346 keV) of the ^68^ZnO(PE) sample in Fig. 14b (and those of the ^68^ZnO(Pb) sample) are much larger than those of the ^68^ZnO(no) sample in Fig. 14a. We also found that the γ-ray intensities for the metallic ^68^Zn(PE) sample in Fig. 14c were much smaller than those of the oxide ^68^ZnO(PE) sample in Fig. 14b. However, the γ-ray intensities of ^67^Cu, ^65^Ni, and ^65^Zn of the ^68^ZnO(PE) and ^68^Zn(PE) samples were approximately the same as those of the ^68^ZnO(no) sample.

The activities for various isotopes of the ^68^ZnO and ^68^Zn samples at the end of irradiation (EOI) were obtained, as shown in Table 9, where the corresponding nuclear reaction path and the reaction threshold energies are also indicated. Here, as an example, in order to know the dependence of the activities for the ^68^ZnO(PE) sample on *d* we took the difference between the activities for the ^68^ZnO(PE) and for the ^68^ZnO(no) samples and divided this difference by the activity for ^68^ZnO(no) (columns F and G for *d* = 3 and 6 cm, respectively, in Table 9). The enhancement factors for *d* = 3 and 6 cm are compared by taking their ratio (column H in Table 9).

The results given in Table 9 are summarized in terms of the activity A(X) of a particular radioisotope X at the end of irradiation (EOI), as follows. Firstly, A(^67^Ga), A(^66^Ga), A(^69m^Zn), and A(^64^Cu) obtained for the ^68^ZnO(PE) sample placed at *d* = 3 cm are more than twenty-times larger than those for the ^68^ZnO(no) sample (column F). Their yields of the ^68^ZnO(PE) sample placed at *d* = 6 cm are about one-fourth of those for the sample at *d* = 3 cm, indicating a strong dependence of the yields on *d*. Secondly, their yields of the metallic ^68^Zn(PE) sample at *d* = 3 cm (column E) are approximately 1/100, 1/100, 1/50, and 1/50 of those of the ^68^ZnO(PE) sample at *d* = 3 cm, respectively. Thirdly, A(^67^Cu), A(^65^Ni), and A(^65^Zn) of the ^68^ZnO(PE) and ^68^Zn(PE) samples are approximately the same as those of the ^68^ZnO(no) sample within an uncertainty factor of 2.

Based on these findings we consider that they indicate a main reaction process to generate protons and neutrons, which play a key role in the large productions of ^67^Ga, ^66^Ga, ^64^Cu, and ^69m^Zn. The second part of the summary, their smaller yields at *d* = 6 cm, suggests that they might be generated by any interaction between primary neutrons (hereafter n_prim_) produced by the Be(*d*,*n*) reaction and nuclei in the polyethylene blocks. On the other hand, the third part of the summary indicates that these protons and neutrons should be dominantly generated in the ^68^ZnO(PE) sample not in the polyethylene blocks via some type of nuclear interaction between neutrons scattered backwards by the polyethylene blocks (hereafter n_sc_) and oxygen nuclei in the ^68^ZnO sample. The anomalous large yields are considered to be dominated by n_sc_. This is apparently unexpected, since the flux of n_sc_ is much reduced compared with that of n_prim_, and the energy of n_sc_ is lower than that of n_prim_. When the energy of n_sc_ is low, that of protons produced by any reaction process between n_sc_ and nuclei in the samples is also low. The cross sections of the ^68^Zn(*p*,2*n*)^67^Ga reaction with a threshold energy of 12.2 MeV and the ^68^Zn(*p*,3*n*)^66^Ga reaction with a threshold energy of 23.6 MeV decrease with a proton energy of less than about 26 MeV.^55)^

Next, we studied the neutron energy dependence of anomalous large yields observed at *E*_d_ = 50 MeV by using 40 MeV deuterons. Enriched ^68^ZnO samples with and without polyethylene blocks were irradiated with 40 MeV deuterons. Note that the neutron energy from 40 MeV deuterons is smaller than that from 50 MeV deuterons. The measured activities of ^67^Cu, ^69m^Zn, and ^64^Cu for the ^68^ZnO sample are given in Table 10. Contrary to the results for 50 MeV deuterons, the yields of the observed radioisotopes for 40 MeV deuterons were independent of the existence of polyethylene blocks. It is evident that the primary-neutron energy played a key role in the large yields of the various radioisotopes mentioned above.

#### 3.3.2 Estimation with Particle and Heavy Ion Transport code System (PHITS).

We calculated the produced activities, A(X), for ^68^ZnO(no) and ^68^ZnO(PE) samples for 50 and 40 MeV deuterons to compare with the measured activities. The calculation was performed using the PHITS code with the geometry of the experimental setup shown in Fig. 13, and the evaluated production cross section data of neutron- and proton-induced reactions given in the fourth version of the Japanese Evaluated Nuclear Data Library (JENDL-4.0/HE). The neutron production rates from the ^9^Be(*d*,*n*) reaction at *E*_d_ = 50 and 40 MeV were determined so that the PHITS simulation gives the same angular and energy distributions of neutrons as those of the latest data reported by Meulders *et al.*^56)^ and Saltmarsh *et al.*^57)^ Note that the neutron data at *E*_d_ = 50 MeV and at *E*_d_ = 40 MeV were obtained by using 10-mm-thick and 6.3-mm-thick Be targets, respectively. We used a 9-mm-thick Be target for 50 and 40 MeV deuterons. Hence, we corrected for any difference in the attenuation of the neutron fluence inside the Be target between the previous studies and the present study by using the PHITS code.

As given in Table 10, the calculated activities of ^67^Cu, ^67^Ga, ^66^Ga, ^69m^Zn, and ^64^Cu from the ^68^ZnO(no) sample for 50 and 40 MeV deuterons are consistent with the measured ones within a factor of 3. Here, ^67^Cu are produced by the ^68^Zn(*n*,*x*) reaction. ^67^Ga and ^66^Ga are generated by the ^68^Zn(*p*,2*n*) and ^68^Zn(*p*,3*n*) reactions in which protons that passed the Be target after the deuteron breakup reaction on Be are considered to be bombarded on the ^68^ZnO(no) sample. The ^68^Zn(*p*,*n*α)^64^Cu and ^64^Zn(*n*,*p*)^64^Cu reactions contribute to generate ^64^Cu. Note that the isotopic composition of ^64^Zn in the ^68^ZnO sample is 0.047 at.%. Contrary to the cases for the ^68^ZnO(no) sample, the calculated activities of ^67^Ga, ^66^Ga, and ^69m^Zn were smaller by a factor of about 15 to 75 than the measured ones, as given in Table 11, and almost the same as those obtained from the ^68^ZnO(no) sample. The reason why the measured activities differ from the calculated ones is unclear at present. Some sort of nuclear reaction process between oxygen nuclei in the ^68^ZnO sample and the scattered neutrons could be the origins of the discrepancy.

In conclusion we discovered anomalously large yields of ^67^Ga, ^66^Ga, ^69m^Zn, and ^64^Cu by irradiating a ^68^ZnO sample that was covered with polyethylene blocks with neutrons provided from the ^9^Be(*d*,*n*) reaction by 50 MeV deuterons, but not by 40 MeV deuterons. This finding will be important for the production of radioisotopes in large quantity with accelerator neutrons by using not only incident accelerator neutrons on a sample, but also neutrons scattered backward from polyethylene blocks simultaneously. Especially, we could produce several radioisotopes which are normally generated by proton-induced reaction from a single sample irradiated with the backscattered neutrons.

## 4 Separation

### 4.1 Separation of ^99m^Tc from ^99^Mo.

The specific activity of fission-^99^Mo is high, ∼370 TBq/(g Mo),^37)^ and therefore ^99m^Tc with high specific activity that is sufficient for performing medical diagnostics is recovered by using quite small volumes of saline with high separation efficiencies of 80 to 90% under repeated elution (milking) by using a ^99^Mo/^99m^Tc generator. In fact, about 1 ml of saline is enough to recover ^99m^Tc with an activity of 740 MBq. On the other hand, a typical specific activity of ^99^Mo produced by any alternative production method of ^99^Mo without the fission reaction of ^235^U is very low, about 1/5,000 of fission-^99^Mo.^22)^ Hence, when one uses the ^99^Mo/^99m^Tc generator, about 5 l of saline is required to collect ^99m^Tc of 740 MBq, which would result in an unacceptably low concentration of eluate for the direct formulation of radiopharmaceuticals. Note that a typical activity of a solution of ^99m^Tc radiopharmaceuticals administered to a patient is high, about 740 MBq/(a few ml). So far, in order to obtain a high specific activity, ^99m^Tc, from ^99^Mo with such a low specific activity various method, such as chromatographic,^58)^ solvent extraction,^59)^ and thermo-separation methods,^13)^ have been developed.

**Figure 15.**
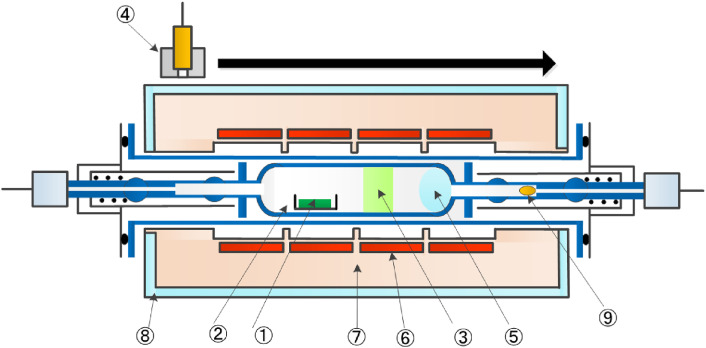
(Color online) Schematic of the experimental setup of the thermochromatographic separation. ① MoO_3_ sample, ② Platinum boat, ③ MoO_3_ needle crystal, ④ CZT detector, ⑤ Quartz wool, ⑥ Heater, ⑦ Heat insulator, ⑧ Water cooling jacket and ⑨ Crumpled gold wire. J. Phys. Soc. Jpn. **84**, 043202 (Ref. 63).

Among these methods, upon considering the following potential we employed the thermo-separation procedure, which utilizes the different volatility of technetium oxide and MoO_3_ to separate ^99m^Tc from ^99^Mo. Namely, with this method it is expected that a large quantity of an enriched ^100^MoO_3_ sample can be used. ^99m^Tc with a high radioactive concentration can be obtained free from any chemical impurities, and an irradiated ^100^MoO_3_ sample can be recovered with high efficiency for recycling. So far, on the basis of thermo-separation, the procedures of sublimation and thermo-chromatography have been developed.^60)^ In sublimation, ^99m^Tc produced in a MoO_3_ powder sample is separated, since it volatilizes at a temperature much lower than the melting point of MoO_3_ at 795 ℃. In thermo-chromatography, Tc and Mo oxides volatilized from a molten MoO_3_ sample are condensed in different temperature zones along a column where the temperature gradient is kept constant in a furnace (see Fig. 15). In reality, before we started to develop a thermoseparation system, the aforementioned potentials of the thermoseparation method had not yet been materialized, owing to the challenges discussed below. In fact, it was not even clear which is better for separating ^99m^Tc from an irradiated Mo sample of over 100 g, a sublimation method or a thermo-chromatography method. We decided to employ the latter by noting a previous study by Tachimori *et al.*^61)^ They first determined a diffusion coefficient, *D*, of ^99m^Tc in MoO_3_ using Fick’s equation of diffusion when considering the release mechanism of ^99m^Tc from MoO_3_ powder samples of 10 to 20 mg. A typical value of *D* was 7.71 × 10^−12^ cm^2^/s at a furnace temperature of 780 ℃.^61)^ Using the *D* at 780 ℃ and Brownian motion theory, the mean diffusion distance, *x*, which is given as *x* = (2*Dt*)^1/2^, was calculated to be ∼10^−4^ cm for one hour of diffusion (*t* = 1 h). Since *D* is so low, we understood that it is impossible to obtain a high separation efficiency of ^99m^Tc from a thick MoO_3_ powder sample which is used for ^99^Mo production by the ^100^Mo(*n*,2*n*)^99^Mo reaction.^62)^ Hence, we employed a thermochromatographic separation method.^62–64)^ A schematic of the experimental setup of the thermochromatographic separation is shown in Fig. 15.

So far, by using the thermochromatographic separation technique many studies were undertaken to measure the separation efficiency by using a molten MoO_3_ sample.^37,65,66)^ However, there remain several challenging problems concerning separation. First is that the separation efficiency of ^99m^Tc, ε_sp_, which is the proportion of ^99m^Tc separated during the thermochromatographic-separation process, is low. This diminishes markedly with repeated sublimation tests (repeated milking tests) at a constant furnace temperature, *T*_fur_, and decreases with an increasing mass of MoO_3_ loaded into a sublimation furnace at a time. For example, the efficiency at furnace loading of a 200 g Mo sample generated via the ^98^Mo(*n*,γ)^99^Mo reaction was 25% on average, and rarely exceeded 50%.^37)^ Note that a milking process of ^99m^Tc from ^99^Mo is usually performed twice per day for at least one week. Typical separation efficiencies of a 5 g MoO_3_ sample were 85, 12, 19 and 24%,^65)^ which were markedly diminished with repeated milking tests. An Idaho group^66)^ used a molten MoO_3_ sample with a thickness of 0.8 mm in order to obtain a high separation efficiency of ^99^Tc, a pure β-emitter. Having such a thin sample is, however, not desirable for the large-scale production of ^99^Mo.

#### 4.1.1 Thermoseparation efficiency of ^99m^Tc from ^99^Mo.

We have challenged these problems by measuring the diffusion coefficient and separation efficiency of ^99m^Tc from a molten MoO_3_ sample of two different thicknesses using a home-made electric furnace, shown in Fig. 15.^62,63)^

**Figure 16.**
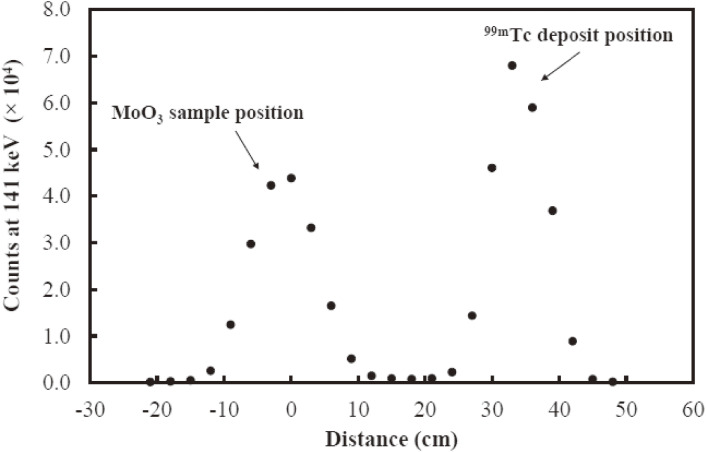
Distribution of the 141 keV γ-ray intensity along a quartz tube measured by the CZT detector after thermochromatographic separation. The vertical axis is the 141 keV γ-ray intensity. The horizontal axis represents the distance from the MoO_3_ sample position along the quartz tube. The deposited ^99m^Tc is clearly separated from the sample. J. Phys. Soc. Jpn. **84**, 043202 (Ref. 63).

A set of three-stage quartz tubes was enclosed by a four-zone tube furnace. A platinum boat was used to hold the irradiated MoO_3_ sample in a high-temperature region throughout the milking. The first two zones were heating sections used to melt the irradiated MoO_3_ sample in a stream of oxygen carrier gas at around 830 ℃ so as to form gaseous materials containing vaporized ^99m^Tc and Mo oxides. The third zone was an intermediate section used to condense any vaporized MoO_3_ as a needle crystal and to transfer gaseous materials containing vaporized ^99m^Tc oxide from the heating sections to a final section. The fourth zone was a final section to collect the separated ^99m^Tc. Since the temperature of the intermediate section was set so as to decrease gradually as the distance in the intermediate section from the heating section increases, the temperature of the gaseous materials at the final section became sufficiently low so as to produce condensation of the gaseous ^99m^Tc products. A quartz wool filter was placed within the tube at a temperature below the melting point of MoO_3_ so as to stop the migration of any volatilized MoO_3_ towards the final section. Crumpled gold wire was placed in the tube to increase the surface area as much as possible for ^99m^Tc collection. The distribution of the ^99m^Tc activity along the tube was investigated using the cadmium zinc telluride (CZT) detector; its peak activity was in the condense region, clearly separated from the ^100^MoO_3_ sample, as shown in Fig. 16.

**Figure 17.**
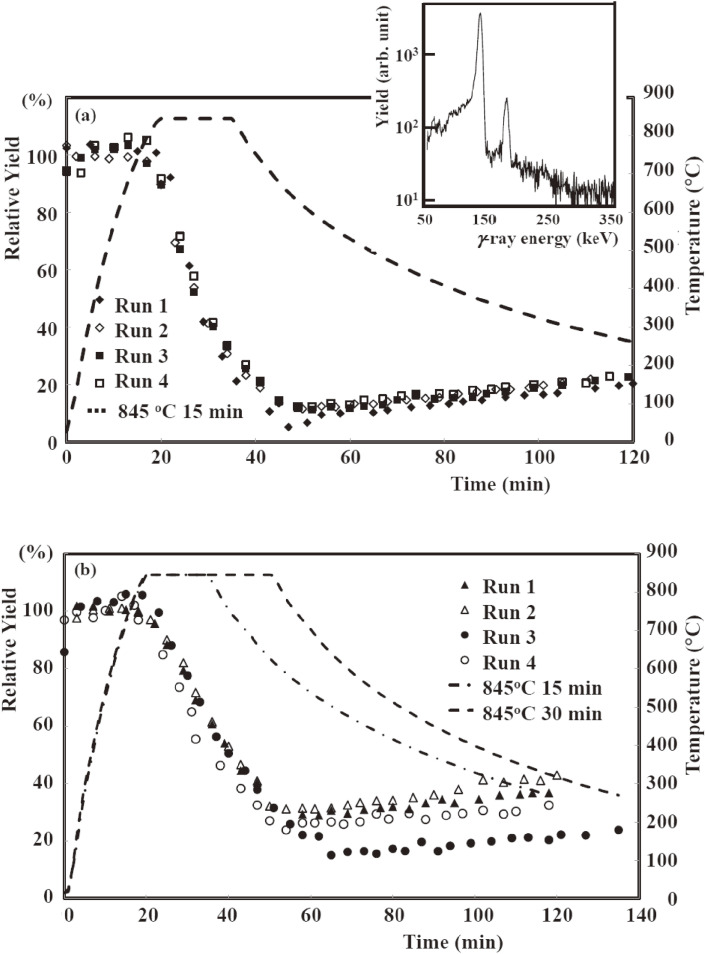
(a) The 141 keV γ-ray yields of ^99m^Tc milking processes obtained by the CZT-1 detector for a 4.0 mm thick MoO_3_ sample at *T*_fur_ = 845 ℃ for *t*_heat_ = 15 min. Both the 141 and 181 keV γ-rays from ^99m^Tc and ^99^Mo are clearly observed (inserted). Four milking runs (filled diamonds, open diamonds, filled squares, and open squares) are shown. (b) The γ-ray yields of ^99m^Tc for the 8.5 mm thick MoO_3_ sample at *T*_fur_ = 845 ℃ for *t*_heat_ = 15 min {Run 1 (filled triangles), Run 2 (open triangles)} and for *t*_heat_ = 30 min {Run 3 (filled circles), and at *T*_fur_ = 855 ℃ for *t*_heat_ = 15 min {Run 4 (open circles)}. The dotted and dashed lines indicate the furnace temperature at *T*_fur_ = 845 ℃ for *t*_heat_ = 15 min and for *t*_heat_ = 30 min, respectively. The γ-ray yield in the time range between 0 and 18 min was normalized to 100%. J. Phys. Soc. Jpn. **83**, 083201 (Ref. 62).

A sequential milking process used to separate ^99m^Tc from molten ^99^MoO_3_ samples of 4.0 and 8.5 mm thicknesses was carried out every for ∼24 hours at a furnace temperature (*T*_fur_) of *T*_fur_ = 845–855 ℃ for a heating time (*t*_heat_) of 15–30 min. We used two CZT γ-ray detectors to study the separation efficiency and the diffusion coefficient of ^99m^Tc in a molten MoO_3_ sample by detecting the 141 keV and 181 keV γ-rays from the decays of ^99m^Tc and ^99^Mo, respectively. The separation efficiency was determined by comparing the 141 keV γ-ray yield of the separated ^99m^Tc with that of the untreated ^99m^Tc within the irradiated MoO_3_ sample before the separation. CZT detectors were placed at the quartz boat (CZT-1) and quartz wool (CZT-2) positions, respectively. The 141 γ-rays yields obtained by the CZT-1 detector for the 4.0 and 8.5 mm thick samples during the milking processes are shown in Figs. 17a and 17b, respectively. For the first 18 min, *i.e.*, before melting of the samples, the γ-ray yield was normalized to 100%. A marked release of ^99m^Tc, which then condensed on the inner wall of the cooled quartz tube, was observed, after the furnace temperature reached the melting point of MoO_3_ at 795 ℃ after *t* ≈ 20 min. The deposition of ^99m^Tc that thermally diffused through the quartz tube was confirmed by CZT-2. It should be noted that all of the 141 keV γ-ray yields of a 4.0 mm (8.5 mm) thick sample obtained by CZT-1 showed the same dependence on the temperature and time, which indicates that the high separation efficiencies of ^99m^Tc remained constant during the sequential milking processes, which differed from the previously reported results. The 141 keV γ-ray was observed after the release of ^99m^Tc from the molten MoO_3_ sample, which included γ-ray emission from the 141 keV state of ^99^Tc fed by γ-decay from the 181 keV state, which was populated by the decay of ^99^Mo, not via the 143 keV state (^99m^Tc) (see Fig. 1), as well as the remaining ^99m^Tc in the sample.

The separation efficiency of ^99m^Tc, ε_sp_, was derived by comparing the 141 keV γ-ray intensity obtained by the CZT-1 detector after separation, *Y*_sep_, with that before the separation *Y*_unt_. *Y*_sep_ and *Y*_unt_ are given as the sum of the intensities of the 141 keV γ-ray emitted from decays of the 143 keV state (^99m^Tc), *Y*(Tc), and the 181 keV state: *Y*(Mo). Using the Bateman equation for the parent-daughter decay, *Y*(Tc) and *Y*(Mo) are given as follows:

$$Y_{\text{unt}}=[Y\text{(Mo)}+Y\text{(Tc)}],$$ [2]

$$Y_{\text{sep}}=[Y\text{(Mo)}+(1-\varepsilon_{\text{sp}})\times Y\text{(Tc)}],$$ [3]

$$Y\text{(Mo)}=A{\text{(Mo)}}_{0}\times \exp(-\lambda_{1}t)\times 0.047,$$ [4]

$$\begin{array}{rl} Y\text{(Tc)} & =[0.88\times \lambda_{2}\times (\lambda_{2}-\lambda_{1}{)}^{-1}\times A{\text{(Mo)}}_{0}\\ & \;\times \{\exp(-\lambda_{1}t)-\exp(-\lambda_{2}t)\}]\times 0.89, \end{array}$$ [5]

where *A*(Mo)_0_ is the ^99^Mo activity at *t* = 0; λ_1_ and λ_2_ are the decay constants of ^99^Mo and ^99m^Tc, respectively. In Eq. [4], the last number of 0.047 comes from the intensity of the 40 keV transition from the 181 keV state to the 141 keV one, 0.053, and the total internal conversion coefficient of the 141 keV transition, 0.119; hence, the 141 keV γ-ray transition intensity is 0.053/1.119 = 0.047.^62)^ In Eq. [5], the first 0.88 in the brackets comes from the fact that 88% of the parent ^99^Mo decays to ^99m^Tc, and the last 0.89 outside the brackets is obtained by taking account of the 141 keV transition intensity per disintegration of ^99m^Tc and the total internal conversion coefficient of the 141 keV transition. Hence, the ratio R of *Y*_sep_ to *Y*_unt_ is given as follows:

$$\begin{array}{rl} R & =\frac{Y_{\text{sep}}}{Y_{\text{unt}}}\\ & =\frac{\left[\exp(-0.0105t)\times 0.047+(1-\varepsilon_{\text{sep}})\times 0.85\times \{\exp(-0.0105t)-\exp(-0.1155t)\}\right]}{\left[\exp(-0.0105t)\times 0.047+0.85\times \{\exp(-0.0105t)-\exp(-0.1155t)\}\right]}. \end{array}$$ [6]

Using Eqs. [2] to [6], high separation efficiencies of close to 90 and 70% on average were obtained for molten MoO_3_ samples of 4.0 and 8.5 mm thickness, respectively, in repeated milking processes at *T*_fur_ = 845 ℃ for *t*_heat_ = 15 min, as given in Table 12.^62)^

The diffusion coefficient, *D*, of ^99m^Tc within the molten MoO_3_ samples was derived as follows. So far, the *D* value of a radioactive ion in a molten sample had not yet been obtained by a release measurement of the ion. In this study, by referring to the work on foil targets for the production of radioactive ion beams at the Isotope Separator On-Line (ISOLDE) at CERN,^67)^ we divided the release process of ^99m^Tc into the following two steps: 1) the transport of ^99m^Tc to the surface of the molten MoO_3_ sample, and 2) the evaporation of ^99m^Tc from the surface of the molten MoO_3_ sample. In Ref. 18 the *D* of radioactive ions in metal targets, which were heated to high temperatures, was determined by assuming that step 2) can be neglected. Based on the same assumption, and by referring to work on the self-diffusion of radioactive ions in a sodium tungstate solution using ^187^W (*T*_1/2_ = 24 h) with a capillary method,^68)^ in which the evaporation process of ^187^W was not included, the diffusion coefficient of ^99m^Tc could be derived. A fraction of the original amount of ^187^W, which is left in a capillary cell at the end of the diffusion, δ, is given using Fick’s law of diffusion:

$$\delta \approx (8/\pi^{2})\times e^{-\theta },$$ [7]

where the parameter θ is given by θ = π^2^*Dt*/(4*h*^2^), *t* is the time of diffusion, and *h* is the length of the capillary cell.

Using Eq. [7] and neglecting the evaporation process of ^99m^Tc, the averaged values of *D*, 〈*D*〉, of ^99m^Tc for the molten MoO_3_ samples with 4.0 and 8.5 mm thicknesses could be derived, as presented in Table 12, where 〈*D*〉 is given by correcting the change of the sample thickness during the repeated milking process. The 〈*D*〉 value of about 1 × 10^−4^ cm^2^/s is much larger than a reported value of 7.71 × 10^−12^ cm^2^/s at 780 ℃ for a powder sample.

In this study high separation efficiencies of about 90 and 70% were successfully obtained through a repeated milking process by the thermo-separation of ^99m^Tc from 10 and 14 g molten MoO_3_ samples with thicknesses of 4.0 and 8.5 mm. By further developing the thermo-chromatography separation system we achieved a higher separation efficiency of over 90% for an irradiated MoO_3_ sample of about 100 g. In these studies, the irradiated MoO_3_ samples were melted in every milking process, and therefore the process could be performed under the same condition as that of the MoO_3_ sample irrespective of the number of processes.

The diffusion coefficients of ^99m^Tc were found to be very large, and therefore ^99m^Tc could diffuse very rapidly in a thick molten sample within a reasonable heating time of 15 to 30 min, and subsequently evaporate from the sample. The present result solves the long-standing problems concerning the thermo-separation of ^99m^Tc from a MoO_3_ sample with an increase in the sample mass or with repeated sublimation, and will bring a major breakthrough in the production of high-quality ^99m^Tc by using a massive ^100^Mo sample.

#### 4.1.2 Quality test of ^99m^Tc from ^99^Mo separated by thermochromatography.

Here, we discuss the safety and efficacy of the “desired” ^99m^Tc radiopharmaceutical to assure parenteral administration to a patient. The United States Pharmacopeia (USP) contains regulatory requirements concerning the radionuclide purity and the radiochemical purity of ^99m^Tc and the concentration of aluminum (Al) in the ^99m^Tc product used to prepare radiopharmaceuticals.^69)^ Namely, the amount of ^99^Mo and the total concentration of all other β^−^ and γ-ray emitters in the ^99m^Tc product must be less than 0.015% and 0.01%, respectively. The radiochemical purity of ^99m^TcO_4_^−^ (pertechnetate) in a saline solution must be above 95% and the chemical purity of ^99m^Tc must be above 90%. Note that chemical impurities generate “undesired” ^99m^Tc compounds, such as free ^99m^TcO_4_^−^, which does not bind to a ligand and thus do not accumulate in a targeted organ of a patient, and thus lead to an extra radiation dose to non-targeted organs of the patient and to cause serious errors in diagnosis. The Al concentration must be less than 10 ppm. Endotoxin, known to be a pyrogen, is another important item, since even small amounts of endotoxin can cause illness in humans. The USP sets the maximum endotoxin concentration limit to be 175 EU/V, where EU is endotoxin units and V is the maximum recommended total dose in milliliters (mL).

Currently, ^99m^TcO_4_^−^, which meets the USP requirements, is obtained from a ^99^Mo/^99m^Tc generator, in which the fission-^99^Mo is loaded to an alumina column and ^99m^Tc in the form of ^99m^TcO_4_^−^ is repeatedly eluted from the column in a saline solution. Chemical impurities, which inhibit the labeling of the ^99m^Tc radiopharmaceutical complex, generate “undesired” ^99m^Tc compounds, such as free ^99m^TcO_4_^−^ and hydrolyzed-reduced ^99m^Tc.

It should be noted that the current USP sets those pharmacopeia standards for ^99m^TcO_4_^−^ obtained from a ^99^Mo/^99m^Tc generator, but there are no pharmacopeia standards for ^99m^TcO_4_^−^, which is obtained by a ^99^Mo (or ^99m^Tc) production method other than one with fission-^99^Mo.^3)^ Therefore, it is important to investigate the pharmaceutical equivalence of ^99m^TcO_4_^−^ to that obtained from the alumina-based ^99^Mo/^99m^Tc generator. In fact, such a study has been conducted concerning the production of ^99m^Tc by the ^100^Mo(*p*,2*n*)^99m^Tc reaction^70)^ and ^99^Mo by the ^100^Mo(γ,*n*)^99^Mo reaction,^71)^ but it has not yet been performed in the production of ^99m^Tc by the ^100^Mo(*n*,2*n*)^99^Mo reaction. Hence, we have tested the pharmaceutical equivalence of ^99m^TcO_4_^−^ obtained by ^99m^Tc from the thermochromatographic separation procedure to that obtained from the alumina-based ^99^Mo/^99m^Tc generator. In addition, we have studied quality-control specifications associated with new variables, such as the contamination arising from an enriched ^100^Mo sample. The USP does not set any criteria concerning the nonradioactive (stable) Mo content in ^99m^Tc-radiopharmaceuticals because an enriched ^235^U sample, which does not contain a stable Mo, and the ^99^Mo/^99m^Tc generator are used for the production of ^99m^Tc using the fission-^99^Mo. We have adopted a dosage limit of 1,700 µg/day, which is given as the injection agent of stable Mo in a report of the International Conference on Harmonization Guideline for elemental impurities (ICH Q3D),^72)^ which does not cover radiopharmaceuticals, but “is intended to provide guidance for registration applications on the content and qualification of impurities in new drug substances produced by chemical syntheses”. We have also studied quality-control tests of ^99m^Tc-radiopharmaceuticals commonly used for the imaging of brain perfusion (^99m^Tc-ECD), myocardial perfusion (^99m^Tc-MIBI), and kidney (^99m^Tc-MAG3), to ensure the safe clinical use of ^99m^Tc obtained by the ^100^Mo(*n*,2*n*)^99^Mo reaction. The separation of ^99m^Tc from an irradiated ^100^MoO_3_ sample was carried out by the thermochromatographic method,^62,63)^ discussed in chapter 4.1.

The quality-control tests of a ^99m^TcO_4_^−^ saline solution on the radiochemical purity and the radiochemical yields of the ^99m^Tc-radiopharmaceuticals, such as ^99m^Tc-ECD, ^99m^Tc-MIBI, ^99m^Tc-MAG3, and ^99m^Tc-MDP, were performed by paper chromatography and by thin-layer chromatography, respectively. The radionuclide purity was studied by taking a γ-ray spectrum of the purified ^99m^TcO_4_^−^ solution using a high-purity Ge (HPGe) detector. The Al concentration of separated ^99m^Tc was checked by using an Al test paper. Details of preparing ^99m^Tc radiopharmaceuticals using commercially available labelling kits (FUJIFILM RI Pharma Co., Ltd., Japan) are given in Ref. 73. The stable Mo content in a 3 mL ^99m^TcO_4_^−^ saline solution was measured by inductively coupled plasma-atomic emission spectroscopy (ICP-AES). Tests of the endotoxin concentrations were carried out by following the statement that the pyrogenicity of a ^99m^TcO_4_^−^ solution from a particular production procedure should be verified by having a portion of it tested by an independent qualified professional using accepted procedures. The results of the quality assessments of the ^99m^TcO_4_^−^ saline solution and ^99m^Tc-radiopharmaceuticals were shown to satisfy the USP requirements listed in Table 13.^73)^ The endotoxin concentrations were below the limit of detection (0.03 EU/mL), much less than the established limit in pharmacopoeias. The measured maximum value of the stable Mo content was 0.138 ppm, *i.e.*, 0.138 µg/mL, which is much less than the permitted daily exposure of 1,700 µg/day given in the ICH Q3D guideline. These results provide important evidence that ^99m^Tc prepared by thermochromatographic separation using ^99^Mo produced by the ^100^Mo(*n*,2*n*)^99^Mo reaction can be a promising substitute for the fission product ^99^Mo.

#### 4.1.3 Recovery of an irradiated ^100^MoO_3_ sample.

It is important to recycle an enriched (expensive) ^100^Mo sample irradiated by neutrons, because a loss fraction of the quantity of the ^100^Mo sample of 100 g during neutron irradiation for 24 h is estimated to be small, about 0.0001%. Here, accelerator neutrons are assumed to be produced by the ^nat^C(*d*,*n*) reaction using 40 MeV, 2 mA deuteron beams, similarly to the estimation given in Table 5. We have therefore developed a recovery method of ^100^MoO_3_ having a recovery efficiency, ε_rec_, higher than 99% to mitigate the cost of a ^100^MoO_3_ sample loss.^74)^ So far, the recovery efficiency, ε_rec_, of the ^100^MoO_3_ samples in the range of 84–97% has been reported in a study of the thermochromatography of ^94m^Tc (*T*_1/2_ = 52 min) from ^94^MoO_3_^75)^; a recovery efficiency of 87%^66)^ or 90%^76)^ was obtained in a ^99^Mo or ^99m^Tc production study based on the ^100^Mo(γ,*n*)^99^Mo or ^100^Mo(*p*,2*n*)^99m^Tc reactions by employing a chemical process.

**Figure 18.**
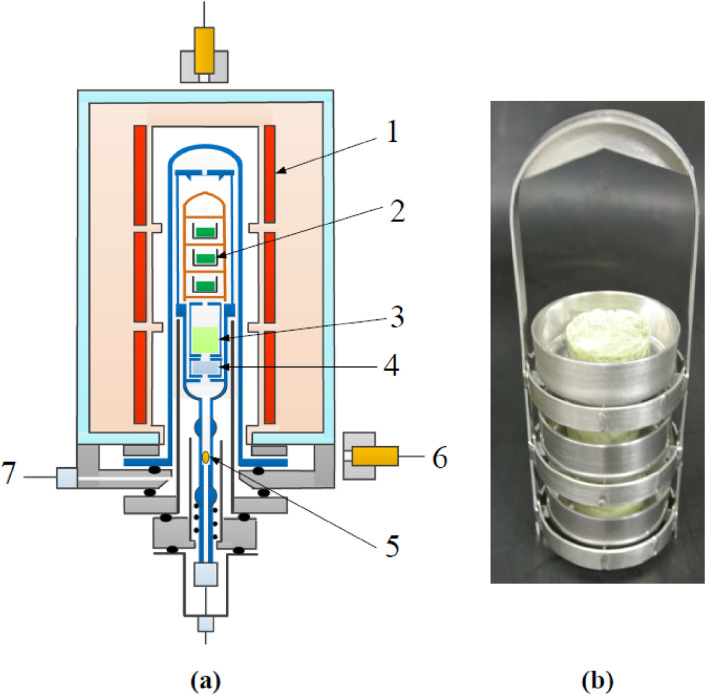
(Color online) (a) Schematic view of thermochromatography system. 1, Electric furnace (three individual heating zones); 2, Crucibles for MoO_3_ samples; 3, MoO_3_ needle crystal holder; 4, Quartz wool; 5, ^99m^Tc condensation region; 6, CZT detector; 7, Moist oxygen inlet. (b) Photograph of sample shelf holding three crucibles with ^100^MoO_3_. J. Phys. Soc. Jpn. **86**, 053201 (Ref. 74).

The present recovery test was performed using a home-made electric furnace, shown in Fig. 18.

An irradiated enriched ^100^MoO_3_ sample of 26.450 g was divided into three platinum crucibles in a vertical three-zone tubular electric furnace along with 103.253 g of non-neutron-irradiated ^100^MoO_3_ to bring the sample mass up to 129.703 g so as to develop a recovery method of over 100 g of ^100^MoO_3_. After the milking process was carried out eight times in total, ^100^MoO_3_ was recovered in two batches from the first five and the following three milking processes. The recovery was focused on the two main deposition sources (the sample remaining in crucibles and needle crystals) in the first batch (run 1), and the detailed distribution of ^100^MoO_3_ was investigated in the second batch (run 2), including in the quartz tube, the platinum shelf, and the quartz wool. The recovery was studied gravimetrically by measuring the weight of ^100^MoO_3_ remaining in the crucibles and being deposited onto any quartz pieces that ^100^MoO_3_ could travel through. All of the crucibles and quartz pieces were weighed before and after the thermochromatography to determine the amount of recovered ^100^MoO_3_ free of any possible contamination caused by the process. Firstly, the ^100^MoO_3_ sample remaining in the crucibles was melted at 830 ℃ in an electric furnace and collected into a quartz test tube using a funnel. Secondly, the ^100^MoO_3_ needle crystals were collected by washing them off the holder with pure water, and then evaporated to dryness to measure the weight. Thirdly, the quartz wool that trapped ^100^MoO_3_ crystals was heated to above 830 ℃, and thereby the ^100^MoO_3_ alone was separated from the quartz wool by thermochromatography. Any quartz pieces, including the funnel used to channel molten ^100^MoO_3_ into the quartz test tube, were washed with pure water in an ultrasonic bath to recover the most ^100^MoO_3_ possible in run 2. The collected ^100^MoO_3_ crystals were evaporated to dryness to measure the weight.

The distribution and recovery yield of the ^100^MoO_3_ mass after thermochromatography is summarized in Table 14. Concerning run 1, after thermochromatography, 118.870 g (92%) out of the initial ^100^MoO_3_ mass of 129.703 g was found to remain in the crucibles, while 9.477 g (87%) out of the 10.833 g of ^100^MoO_3_ that was vaporized from the crucibles was trapped as needle crystals. During the recovery process of 118.870 g of ^100^MoO_3_ in the crucibles, a certain amount of ^100^MoO_3_ was vaporized and then trapped as needle crystals. Hence, the recovery from the needle crystals was 10.296 g, which was more than the needle crystal mass of 9.477 g measured after thermochromatography. Finally, 117.502 g (99%) out of 118.870 g of ^100^MoO_3_ was recovered from the crucibles and 10.296 g from the needle crystal holder, which gave a recovery yield of 98.5% (127.798/129.703 = 0.985). For run 2, 110.551 g (94%) out of the initial ^100^MoO_3_ mass of 117.490 g was found to remain in the crucibles after thermochromatography. The vaporized ^100^MoO_3_ from the crucibles (6.939 g) was deposited onto the quartz tubes (0.073 g), quartz wool (0.376 g), and a needle crystal holder (6.380 g), and was 6.829 g in total. The recovered ^100^MoO_3_ from the crucibles was 108.640 g (92%), and that from other than the crucibles was 8.426 g, giving a total recovery of 117.066 g (99.6%). Note that 1.911 g of ^100^MoO_3_ was vaporized from the crucibles and then trapped as needle crystals during the recovery process, which resulted in the total amount of recovered ^100^MoO_3_ deposited, other than in the crucibles (8.740 g) being larger than the amount of needle crystals measured after the separation.

A high recovery yield of 99% was obtained, which would significantly reduce any financial damage due to the loss of the enriched ^100^MoO_3_ sample. We consider that the newly developed home-made thermochromatography system should have a capability of nearly 100% recovery, because all of the ^100^MoO_3_, including the small unrecovered amount, is kept within the thermochromatographic apparatus inside the electric furnace.

### 4.2 Separation of ^64^Cu and ^67^Cu and biodistribution of ^67^CuCl_2_ in tumor bearing mice.

#### 4.2.1 Separation of ^64^Cu and ^67^Cu from Zn.

**Figure 19.**
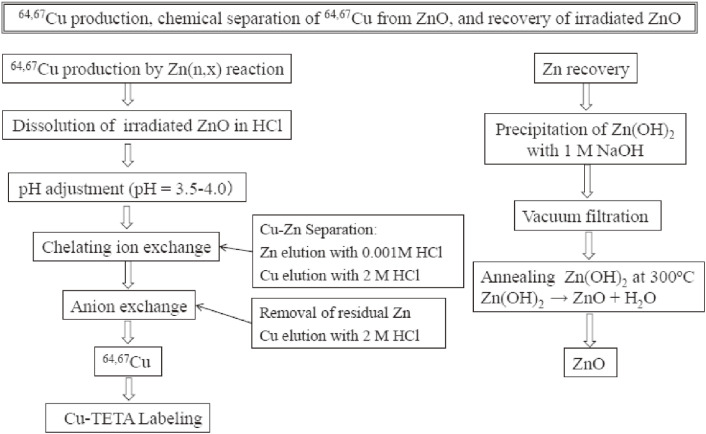
Flow chart of ^64,67^Cu separation from irradiated ZnO samples and the recovery of ZnO. J. Radioanal. Nucl. Chem. **303**, 1205–1209 (Ref. 77).

We have developed a radiochemical separation of ^64^Cu and ^67^Cu produced by the ^64^Zn(*n*,*p*)^64^Cu, ^67^Zn(*n*,*p*)^67^Cu and ^68^Zn(*n*,*x*)^67^Cu reactions using ^nat^ZnO or ^64^ZnO samples.^77)^ A flowchart of the separation steps of the irradiated samples is given in Fig. 19.

The irradiated sample of 5.225 g was dissolved in 20 ml of 36 wt% HCl, which was passed through an ion-exchange column for adsorbing the Cu ions, and thus separating Zn. The ^64,67^Cu was then eluted with 20 ml of 2.0 M HCl, passed through an anion-exchange column to remove traces of Zn, followed by washing with 10 ml 2.0 M HCl to obtain purified ^64,67^Cu radionuclides. The collected efficiency of ^64^Cu separated from the irradiated ^64^ZnO sample was 96%. The time required for the column separation process was 3–4 h. We also developed a method for recycling irradiated enriched ^64^Zn and ^68^Zn samples after radiochemical separation. The recovery efficiency of the ^nat^ZnO sample was demonstrated to be over 95% in a cold (non-radioactive) run using an alkaline precipitation method. The purified ^64,67^Cu solution was reacted with a bifunctional ligand used for antibody labelling. The labelling yield was determined by thin-layer chromatography (TLC) to be good at 92–97% which was satisfactory for clinical radiotherapy applications.

#### 4.2.2 Biodistribution of ^67^CuCl_2_ in tumor-bearing mice.

We developed new production routes to improve the low-availability of the promising radionuclide of ^64^Cu and ^67^Cu, and to establish a radiochemical method for obtaining high-quality ^64^Cu and ^67^Cu from neutrons irradiated Zn samples. Cu-based radiopharmaceuticals that can accumulate in cancer cells, such as ^64^Cu-labeled proteins, peptides, and antibodies, have been developed and widely used.^4)^ However, currently ^64^Cu complexes are considered to have relatively low stability *in vivo*, which could cause the loss of ^64^Cu from the complexes, leading to less accumulation of ^64^Cu in targeted cancer cells by producing free radioactive ^64^Cu.^5)^ ^64^Cu chloride (^64^CuCl_2_) has been identified as a potential agent for PET imaging and radionuclide therapy.^78)^ In a study using ^64^CuCl_2_ relevant to radionuclide therapy, it was demonstrated that Cu metabolism is important for many cancers. Here, it is worth noting that compared with ^64^Cu-labeled complexes, ^64^CuCl_2_ has simple radiochemistry without a radiolabeling process. The results prompted us to measure the biodistribution of ^67^CuCl_2_ in colorectal tumor-bearing mice. Colorectal cancer is a major cause of death in Japan.^79)^

^67^Cu was produced by irradiating an ^68^ZnO (99.935% enriched in ^68^Zn) sample with neutrons at TIARA-QST.^42)^ The chemical separation of ^67^Cu from a neutron-irradiated ^68^ZnO sample was performed by slightly modifying the previously reported method to separate Ga ions.^77)^ The radionuclide purity of ^67^Cu was 99.8% at the time of injection. The specific activity [MBq/(µg Cu)] of ^67^Cu was determined to be 4.5 MBq/(µg Cu) at EOI by the titration method. This value is much smaller than the typical specific activity of ^64^Cu produced by the ^64^Ni(*p*,*n*)^64^Cu reaction in the range of 2.4–11 GBq/(µg Cu) quoted from a recent study on the biodistribution of ^64^CuCl_2_ in rats.^78)^ It is considered that the specific activity plays an important role in radiolabeling and the *in vivo* biodistribution of radioactive tracers. Hence, it is very interesting to study the role of ^67^CuCl_2_ with low specific activity in the biodistribution of ^67^Cu ions in colorectal tumor-bearing mice. Animal procedures were carried out according to a protocol approved by the QST Institutional Animal Care and Use Committee. The ^67^Cu solution after radiochemical purification was diluted with a physiological saline solution for injection into mice. When the tumors were palpable, the mice were intravenously injected with 35 or 50 kBq of ^67^CuCl_2_ dissolved in 100 µl saline via a tail vein. After the mice were sacrificed at 0.5, 1, 4, 8, 24, and 48 h post-injection (*n* = 4, four mice at a time), their blood and organ samples of interest (liver, kidney, intestine without content, spleen, pancreas, stomach, heart, lung, muscle, bone, brain, and tumor) were removed and weighed.^80)^

**Figure 20.**
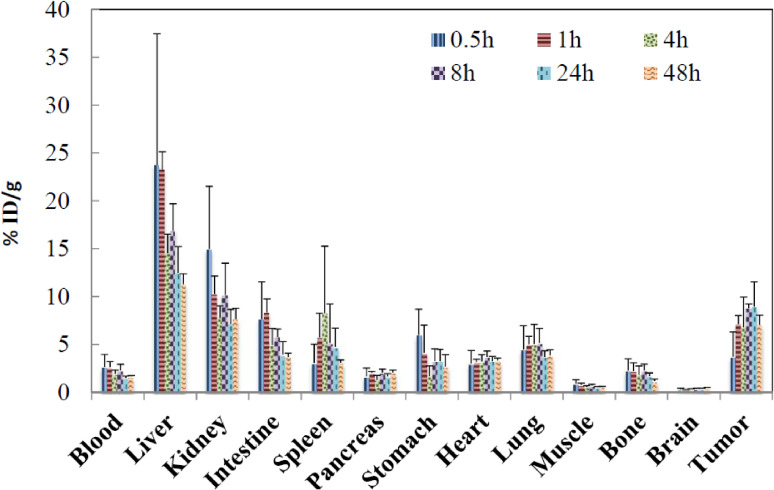
(Color online) Biodistribution of ^67^CuCl_2_ in tumor-bearing mice with standard deviation. J. Phys. Soc. Jpn. **86**, 023201 (Ref. 80).

The radioactivities in the blood and organ samples were measured by using a well-type NaI(Tl) detector. The biodistribution of ^67^CuCl_2_ in tumor-bearing mice was determined, as shown in Fig. 20. Note that it is common in animal studies to express the biodistribution of radiotracers using the parameter %ID/g of tissue, defined as the radioactivity in a particular tissue at each time point as a percentage of the total radioactivity injected into the animal, which was further divided by the weight of each tissue. It is very interesting that a high uptake of ^67^Cu in the tumor was found, which may indicate an important role of Cu metabolism in colorectal cancer. The accumulation of ^67^Cu in the tumor was 7.0 ± 1.4%ID/g at 48 h, comparable to that of ^64^Cu, ∼5%ID/g, in spite of the difference in the specific activities. A high uptake of ^67^Cu was also observed in the organs, such as the liver and kidney. The ^67^Cu uptake in the liver and kidney gradually decreased over time from 0.5 to 48 h. The biodistribution of ^67^CuCl_2_ determined by using very low-specific-activity ^67^Cu is similar to the recent biodistribution of ^64^CuCl_2_ obtained by using high-specific-activity ^64^Cu in malignant melanoma tumor-bearing mice.^78)^ The observed uptake of ^67^Cu in these organs is considered to be due to copper metabolism being independent of the specific activity of ^67^Cu.

In summary, ^67^CuCl_2_ was used for the first time to determine the biodistribution in colorectal tumor-bearing mice. A high uptake of ^67^Cu in the tumor was found, although the specific activity of ^67^Cu was low owing to the neutron intensity currently available. This result suggests that ^67^CuCl_2_ can be a potential radionuclide agent for cancer radiotherapy.

## 5 Deuteron accelerator and neutron source

### 5.1 Deuteron accelerator.

Deuteron beams, provided mostly by linear accelerators, are used to produce high neutron fluxes by irradiating a light element, such as carbon, liquid Li, and Be, for example in the projects SPIRAL2, the Soreq Applied Research Accelerator Facility (SARAF) project in Israel,^81)^ and the International Fusion Materials Irradiation Facility (IFMIF)^82)^ *et al.*, where a fixed neutron energy of 14 MeV is mostly needed. However, in order to produce a particular medical radioisotope by neutron-induced reaction using accelerator neutrons, a wide variety of the neutron energy is necessary, because a neutron-induced reaction cross section on samples depends on the neutron energy. Therefore, the neutron energy needs to be easily tuned to an energy suitable for the production of medical radioisotopes by changing the energy of the deuteron beams. In addition, we will use the same accelerator to produce not only accelerator neutrons, but also proton and deuteron beams, for generating a wide variety of medical radioisotopes via proton- and deuteron-induced reactions on a sample. In considering that accelerators have an active lifespan of over 30 years, and that interest in new medical radioisotopes will be continuously growing, the expected newly installed accelerators must have a capability for producing a wide variety of medical isotopes. We choose AVF cyclotrons with 50 MeV, 2 mA deuteron beams to meet the mentioned requirements. A fixed radiofrequency AVF cyclotron is robust in operation, compact in size, and relatively cheap compared to a linear accelerator. Such cyclotrons can be constructed by many cyclotron companies around the world. In fact, Sumitomo Heavy Industries, Ltd. has been constructing AVF cyclotrons, which can provide a 30 MeV, 1 mA H^−^ beam for Boron Neutron Capture Therapy (BNCT).^83)^ Here, it is worth mentioning that the beam intensities from cyclotrons are limited by an extraction device (deflector), and therefore negative deuteron D^−^ ions should be accelerated up to 50 MeV. The principal advantage of a D^−^ ions cyclotron is the ease and low loss in extraction by the stripping of D^−^ ions into positive deuteron (D^+^) ions on a thin carbon foil with a thickness of about 500 µg/cm^2^. The D^+^ ions can be extracted to a beam line through a residual magnetic field in the AVF cyclotron.

### 5.2 Accelerator neutron source.

So far, various types of accelerator-based neutron sources with high neutron fluxes that have kinetic energy above a few MeV have been developed for fundamental studies in nuclear physics and nuclear astrophysics, radiation-resistant materials irradiation testing for fusion reactors, boron neutron capture therapy, and slow neutron scattering *etc.* In these cases, accelerator neutrons are generated by the ^7^Li(*p*,*n*)^7^Be, ^3^H(*d*,*n*)^4^He, and ^9^Be(*p*,*n*)^9^C reactions, and the spallation reaction by bombarding a liquid mercury target, a liquid bismuth-lead target with high-energy proton beams.

In medical radioisotope productions in reactors, thermal neutron fluxes of a factor × 10^14^ n/(cm^2^ s) have been used. Accelerator neutrons with a quasi-monoenergy of 14 MeV with about 10^12^ n/(cm^2^ s) were used in the field of nuclear engineering at Fusion Neutronics Facility (FNS) of the Japan Atomic Energy Agency (JAEA),^51)^ and at a facility with a rotating target neutron source (RTNS-II) in U.S.A.,^84)^ respectively. Because the ^3^H target is radioactive, and neutrons from the ^3^H(*d*,*n*)^4^He reaction at *E*_d_ = 300 keV are emitted isotopically with respect to the deuteron beam direction, this leads to a disadvantage concerning the effective use of neutrons for RIs productions. Another intense neutron source based on the deuteron breakup reaction was proposed by P. Grand and A. N. Goland.^85)^ Note that Helmholz *et al.* first observed the breakup process,^86)^ and also found that an intense forward-directed beam of neutrons is emitted when a target with a low-atomic number, such as Li or Be, is bombarded with deuteron beams. They also designed a high-flux neutron generator system composed of a 35 MeV high-current deuteron linear accelerator and a molten Li target configuration. Based on the proposed neutron source, at the IFMIF, intense neutron fluxes of greater than 10^15^ n/(cm^2^ s) with the energy spectrum peaking at around 14 MeV are expected to be produced by bombarding a liquid lithium jet target with intense deuteron beams of about 35–40 MeV. At the SPIRAL2 facility, neutrons with a high flux of 10^15^ n/s are planned to be produced by ^nat^C(*d*,*n*) using 40 MeV 5 mA deuterons provided from a linear accelerator. At the SARAF facility, there is an ongoing project with the superconducting light/heavy-ion LINAC, with a potential of about 40 MV, capable of accelerating 5 mA deuterons up to 40 MeV. The neutrons are produced by irradiating deuterons on a liquid Li target.

**Figure 21.**
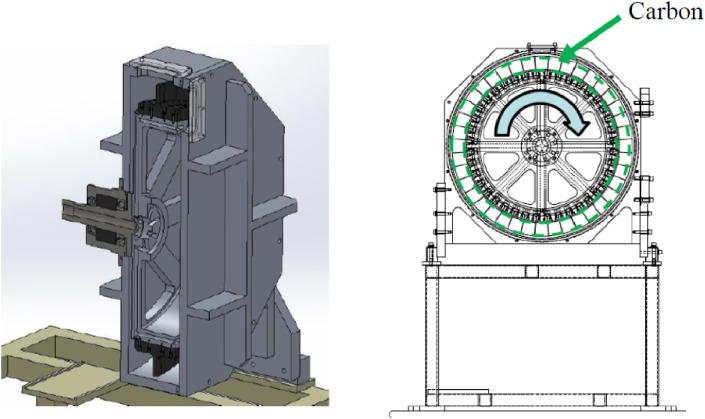
(Color online) Schematic view of the neutron target system.

We have also installed a mini-type rotating carbon target system used for producing accelerator neutrons provided by the ^nat^C(*d*,*n*) reaction in collaboration with Sumitomo Heavy Industries, Ltd. A schematic view of the neutron target is shown in Fig. 21. The carbon target was shown to work successfully under a thermal power of 40 kW using the JAERI Electron Beam Irradiation System (JEBIS) at Japan Atomic Energy Agency (now QST), which can provide 20–100 keV electron beams with an output beam power of 400 kW.^87)^

## 6 Conclusions and future prospects

**Figure 22.**
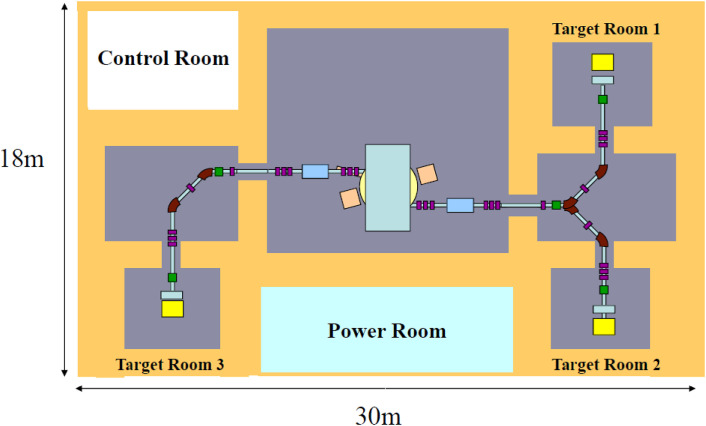
(Color online) Layout of the accelerator facility for radioisotope production. J. Phys. Soc. Jpn. **82**, 064201 (Ref. 46).

We proposed an innovative method to produce a wide variety of medical radioisotopes. By overcoming challenges in the proposed method by fundamental studies, we have successfully carried out all of the important steps necessary to obtain high-quality ^99m^Tc suitable for formulating ^99m^Tc radiopharmaceuticals. In addition, we could validate the high capability of accelerator neutrons so far unexplored to produce a large amount of high-quality therapeutic radioisotopes conducting detailed studies of ^64^Cu and ^67^Cu. In order to secure a constant and reliable supply chain of ^99^Mo for domestic use and to promote a theranostics approach in personalized nuclear medicine, we have presented a proposal for a prototype facility for the Generation of Radioisotopes with Accelerator Neutrons by Deuterons (GRAND).^46)^ The proposed system consists of an AVF cyclotron with a 50 MeV, 2 mA deuteron beam intensity and a rotating carbon target system to produce intense accelerator neutrons. In the cyclotron negative deuteron ions (D^−^) are accelerated up to 50 MeV, and by passing them through a stripper foil, D^−^ beams are converted to D^+^ beams, which are extracted from the cyclotron to a beam-transport system for irradiating a carbon target to produce accelerator neutrons. The principal advantage of a negative deuteron-cyclotron is the ease and low loss in extraction by the stripping of negative deuteron ions into positive deuteron (D^+^) ions on a thin carbon foil with a thickness of about 500 µg/cm^2^. The D^+^ ions can be extracted to two beam lines through a residual magnetic field in the AVF with a possibility to irradiate two different targets simultaneously. The layout of the accelerator is shown in Fig. 22.

In April 2020, the two-year “Deuteron Accelerator for Theranostics mEdicine (DATE)” project at Tohoku University was started. In this project we plan to accelerate 25–40 MeV deuteron beams with an intensity of 100 µA by newly setting up a negative deuterium ion source and a stripper foil for the existing AVF cyclotron at CYRIC at Tohoku University.^34)^ The deuteron beam intensity will be about twenty-times stronger than the presently available D^+^ beam intensity of about 5 µA, and about one-twentieth of the intensity that will be obtained at the GRAND project. At CYRIC, a negative hydrogen beam of 50 MeV with 22 µA was successfully obtained in 2003, which demonstrated the feasibility of high-current acceleration and extraction for proton beams with H^−^ acceleration. The DATE project will play an important role in the on-demand medical RIs production for promoting a theranostics approach in personalized nuclear medicine and also in detailed planning for a prototype facility, GRAND.

## Figures and Tables

**Table 1.** Radioisotopes typically used for nuclear medicine and their production routes. Here, r, g, and a in brackets indicate reactor, generator, and accelerator

Imaging: conventional single photon emission tomography SPECT, SPECT/CT	Imaging: positron emission tomography (PET,PET/CT,PET/MRI)	Therapy, pain palliation, radioimmunotherapeutics	
^67^Ga(a)	^18^F(a)	^67^Cu(a)	^131^I(r)
^99m^Tc/^99^Mo(r)(g)	^61^Cu(a)	^89^Sr(r)	^153^Sm(r)
^111^In(a)	^64^Cu(a)	^89^Zr(a)	^169^Er(r)
^123^I(a)	^68^Ga/^68^Ge(a)(g)	^90^Y/^90^Sr(r)(g)	^177^Lu(r)
^131^I(r)	^82m^Rb/^82^Sr(a)(g)	^90^Y(r)	^186^Re(r)
^133^Xe(r)	^89^Zr(a)	^117m^Sn(r)	^188^Re/^188^W(r)(g)
^201^Tl(a)	^124^I(a)	^123^I(a)	alpha emitters, *e.g.* ^213^Bi/^225^Ac(g)(a)

**Table 2.** Current irradiation facility for producing ^99^Mo around the world. A ‘6-day curie’ is defined as the amount of ^99^Mo activity left six days after the generator has left the producer’s facilities.^19)^ HEU and LEU indicate highly enriched uranium and low enriched uranium used in the fuel of reactors

Reactor	Operating days/year	^99^Mo production weeks/year	Available capacity/year (6-day Ci ^99^Mo) by 2024	Fraction (%)	Expected end of operating
BR-2 (HEU) Belgium	147	21	136,500	17	2026
HFR(LEU) the Netherlands	275	39	241,800	30	2026
LVR-15(LEU) Czech Republic	210	30	90,000	11	2028
MARIA(LEU) Poland	200	36	79,200	10	2040
OPAL(LEU) Australia	300	43	92,450	12	2057
RA-3(LEU) Argentina	230	46	23,000	3	<2027
SAFARI(LEU) South Africa	305	44	130,700	16	2030
^99^Mo demand	46,800		793,650		

**Table 3.** Current ^99^Mo processing facilities in the world

Reactor	^99^Mo production weeks/year	Available capacity/year (6-day Ci ^99^Mo) by 2024	Fraction (%)	Expected end of operating
ANSTO Health Australia	43	92,450	14	2057
CNEA Argentina	46	23,000	3	<2027
IRE Belgium	49	171,500	25	2028
Curium the Netherlands	52	260,000	38	Not known
NTP South Africa	44	130,700	19	2030
Total		677,650		

**Table 4.** Measured ^99^Mo yield (^99^Mo_meas._) at the EOI compared with the calculated yield (^99^Mo_cal._). The number in brackets in the first line is the number of the sample shown in Fig. 6

^nat^MoO_3_ (g)	25.869	25.868	25.483	25.220
	(1)	(2)	(3)	(4)

^99^Mo_meas._(10^4^ Bq)	3.9 ± 0.2	2.6 ± 0.1	1.7 ± 0.1	1.3 ± 0.1

^99^Mo_cal._(10^4^ Bq)	3.6 ± 0.7	2.3 ± 0.4	1.5 ± 0.3	1.0 ± 0.2

**Table 5.** Calculated activities (in units of gigabecquerel) of ^99^Mo at the EOI for an enriched ^100^MoO_3_ sample (100% enriched in ^100^Mo) for 24 h in terms of the radius and thickness of ^100^Mo, and the distance (d) between the natural carbon target and the ^100^Mo sample position

Radius of *d*-beam (cm)	Distance between C and sample (cm)	Radius of sample (cm)	^99m^Tc			^99^Mo		
			100 g	150 g	200 g	100 g	150 g	200 g
0.5	0.5	0.5	1.4E+11	1.4E+11	1.4E+11	2.4E+11	2.4E+11	2.4E+11
		1.0	3.7E+11	3.7E+11	3.8E+11	6.1E+11	6.3E+11	6.4E+11
		2.0	3.3E+11	4.3E+11	4.9E+11	5.5E+11	7.2E+11	8.3E+11
		3.0	1.8E+11	2.6E+11	3.3E+11	3.0E+11	4.3E+11	5.6E+11
	1.0	0.5	1.0E+11	1.0E+11	1.0E+11	1.8E+11	1.8E+11	1.8E+11
		1.0	3.0E+11	3.1E+11	3.2E+11	5.1E+11	5.3E+11	5.3E+11
		2.0	3.0E+11	3.9E+11	4.5E+11	5.1E+11	6.6E+11	7.6E+11
		3.0	1.7E+11	2.5E+11	3.1E+11	2.8E+11	4.1E+11	5.3E+11
	1.5	0.5	8.0E+10	8.0E+10	8.0E+10	1.3E+11	1.3E+11	1.3E+11
		1.0	2.5E+11	2.6E+11	2.6E+11	4.2E+11	4.4E+11	4.5E+11
		2.0	2.7E+11	3.6E+11	4.1E+11	4.6E+11	6.0E+11	6.9E+11
		3.0	1.6E+11	2.3E+11	3.0E+11	2.7E+11	3.9E+11	5.0E+11
	2.0	0.5	6.3E+10	6.3E+10	6.3E+10	1.1E+11	1.1E+11	1.1E+11
		1.0	2.1E+11	2.2E+11	2.2E+11	3.6E+11	3.7E+11	3.8E+11
		2.0	2.5E+11	3.2E+11	3.7E+11	4.2E+11	5.4E+11	6.2E+11
		3.0	1.5E+11	2.2E+11	2.8E+11	2.5E+11	3.7E+11	4.7E+11

**Table 6.** Activities of radionuclides produced by the ^100^Mo(n,2n)^99^Mo reaction. b and Y_γ_ are the γ-ray emission probability and the γ-ray yield, respectively

Nuclides	Reaction	*T*_1/2_ (h)	*E*_γ_ (keV)	ε_γ_ (%)	*b* (%)	*Y*_γ_ (cps)	EOI activity (Bq)
							Measurement
^99m^Tc	^99^Mo decay	6.0	140.5	0.040	89.1	633.1 ± 1.4	—
^99^Mo	^100^Mo(*n*,2*n*)	65.9	181.1	0.036	6.0	53.8 ± 0.4	(3.08 ± 0.11) × 10^6^
			739.5	0.012	12.1	41.5 ± 0.3	(3.21 ± 0.12) × 10^6^
			777.9	0.012	4.3	14.2 ± 0.2	(3.19 ± 0.13) × 10^6^
^97^Zr	^100^Mo(*n*,α)	16.9	743.4	0.012	93.1	2.14 ± 0.09	(31.5 ± 1.6) × 10^3^
^97^Nb	^97^Zr decay	1.2	658.1	0.014	98.4	2.53 ± 0.10	—

**Table 7.** Activity ratios of impurity radionuclides to ^67^Cu produced by ^68^Zn(p,2p)^67^Cu, ^70^Zn(d,αn)^67^Cu, and ^68^Zn(n,x)^67^Cu reactions at EOI together with estimated ones

	Beam energy (MeV)	Ratio to ^67^Cu						
		^64^Cu	^66^Ga	^67^Ga	^69m^Zn	^65^Zn	^65^Ni	^66^Ni
^68^Zn(*p*,2*p*)^67^Cu	100 → 20	10	∼12	∼2.5		∼0.1		
^70^Zn(*d*,α*n*)^67^Cu	19.5 → 18.4	0.1	0.03	0.07	2.3			
Present Exp.		<0.016	0	0	0.14	6.7 × 10^−4^	2.6	(2.4–5.0) × 10^−3^
^68^Zn(*n*,*x*)								
Estimation		7.5 × 10^−4^			0.12	5.1 × 10^−4^	2.9	1.0 × 10^−3^
^68^Zn(*n*,*x*)								
^66^Zn(*n*,2*n*)						1.7 × 10^−4^		
^70^Zn(*n*,2*n*)					0.03			
^64^Zn(*n*,*p*)		8.7 × 10^−3^						

**Table 8.** γ-ray energy of ^67^Cu, ^67^Ga, ^66^Ga, ^69m^Zn, ^64^Cu, ^65^Zn, and ^65^Ni, and the absolute γ-ray branching ratio, I_γ_ (in parenthesis)

	^67^Cu (61.8 h)	^67^Ga (78.6 h)	^66^Ga (9.5 h)	^69m^Zn (13.8 h)	^64^Cu (12.7 h)	^65^Zn (244 d)	^65^Ni (2.52 h)
E_g_ (keV) and *I*_g_ (%)	93 (14.8)	93 (39)	834 (5.89)			1,116 (50.6)	1,116 (15.4)
	185 (44.2)	185 (20.9)	1,039 (37)	439 (94.8)	1,346 (0.47)		1,482 (24)
	300 (0.743)	300 (16.8)					

**Table 9.** Activity (in units of kBq) of radioisotopes produced from the ^68^ZnO(no), ^68^ZnO(PE), ^68^ZnO(Pb), and ^68^Zn(PE) samples at the end of irradiation in columns A–E. Columns F and G give the difference of B, C and A divided by A, and column H is a ratio between G and F. In most cases the uncertainties of measured activities are less than 10%

Radioisotope		^67^Cu	^65^Ni	^65^Zn	^69m^Zn	^67^Ga	^66^Ga	^64^Cu
Reaction		(*n*,*x*)	(*n*,α)	(*n*,4*n*)	(*n*,γ)	(*p*,2*n*)	(*p*,3*n*)	(*p*,α*n*)
E_thr._(MeV)		7.9	0	28.7	0	12.2	23.6	7.9
Sample								
A	^68^ZnO(no)	5.7	21	0.016	3.3	1.5	0.5	0.81
B	^68^ZnO(PE) (*d* = 3 cm)	5.4	33	<0.021	67	65	11	62
C	^68^ZnO(PE) (6 cm)	5.9	23	0.018	14	14	6.9	16
D	^68^ZnO(Pb) (3 cm)	6.1	35	0.022	75	72	15	65
E	^68^Zn(PE) (3 cm)	6.5	19	0.016	1.4	0.6	0.1	0.99
F	(B − A)/A	−0.5	0.6		19	42	20	76
G	(C − A)/A	0.04	0.1		3.2	8.3	12	19
H	G/F = (C − A)/(B − A)	−0.7	0.2		0.2	0.2	0.6	0.25

**Table 10.** Activity (in units of kBq) of radioisotopes produced from ^68^ZnO(no) and ^68^ZnO(PE) samples at the end of irradiations using 40 MeV deuterons. ND = not detected

Radioisotope		^67^Cu	^65^Ni	^65^Zn	^69m^Zn	^67^Ga	^66^Ga	^64^Cu
Reaction		(*n*,*x*)	(*n*,α)	(*n*,4*n*)	(*n*,γ)	(*p*,2*n*)	(*p*,3*n*)	(*p*,α*n*)
E_thr._(MeV)		7.9	0	28.7	0	12.2	23.6	7.9
Sample								
A	^68^ZnO(no)	6.9	ND	ND	1.6	ND	ND	1.2
B	^68^ZnO(PE) (*d* = 3 cm)	8.1	ND	ND	2	ND	ND	1.9

**Table 11.** Measured and calculated activities of radioisotopes produced by an irradiating ^68^ZnO sample without and with polyethylene blocks with neutrons from 50 MeV deuterons (in brackets 40 MeV deuterons). ND = not detected

^68^ZnO(no) sample							
	Activity (kBq) at EOI E_d_ = 50 MeV [40 MeV]						
	^67^Cu	^65^Ni	^65^Zn	^69m^Zn	^67^Ga	^66^Ga	^64^Cu
Exp	5.7 [7.6]	21 [53]	0.016 [ND]	3.3 [1.6]	1.5 [ND]	0.53 [ND]	0.81 [1.2]
Cal	14 [11]	26 [38]	0.045 [0.013]	0.81 [0.88]	3.9 [0.036]	0.74 [0.001]	1.6 [0.12]
Exp/Cal	0.41 [0.69]	0.87 [1.4]	0.35 [ND]	4.1 [1.8]	0.38 [—]	0.71 [—]	0.51 [10]

^68^ZnO(PE) sample							
	Activity (kBq) at EOI E_d_ = 50 MeV						
	^67^Cu	^65^Ni	^65^Zn	^69m^Zn	^67^Ga	^66^Ga	^64^Cu
Exp	5	33	ND	67	73	11	62
Cal	14	26	0.045	0.9	3.9	0.74	1.7
Exp/Cal	0.36	1.27	—	74	19	15	36

**Table 12.** Obtained value of R, the separation efficiency and the diffusion coefficient of ^99m^Tc from molten MoO_3_ samples with 4.0 and 8.5 mm thicknesses for each run. 〈D〉 is the average value of the diffusion coefficient. T_fur_ and t_heat_ are the furnace temperature and the heating time of the furnace, respectively

Run	Sample depth (mm) (Mass (g))	*T*_fur_	*t*_heat_	R	ε_sp_ (%)	*D* (cm^2^ sec^−1^)	〈*D*〉 (cm^2^ sec^−1^)
1	4.0 (9.87)	845	15	0.105	95	7.9 × 10^−5^	6.3 × 10^−5^
2		845	15	0.165	89	6.5 × 10^−5^	
3		845	15	0.163	89	5.8 × 10^−5^	
4	(7.69)	845	15	0.172	88	5.0 × 10^−5^	
1	8.5 (14.3)	845	15	0.333	71	1.4 × 10^−4^	1.4 × 10^−4^
2		845	15	0.354	68	1.4 × 10^−4^	
3		845	30	0.203	84	1.5 × 10^−4^	
4	(11.9)	855	15	0.285	76	1.4 × 10^−4^	

**Table 13.** Results of quality control tests of a ^99m^TcO_4_^−^ saline solution and ^99m^Tc-radiopharmaceuticals along with USP specifications

Parameter	USP	Exp. 1	Exp. 2	Exp. 3	Exp. 4
pH	4.5 to 7.5	7.23	7.16	6.66	6.58
Endotoxins	<175 EU/V	<0.03 EU/mL	>0.03 EU/mL	<0.03 EU/mL	<0.03 EU/mL
Radionuclidic purity - non fission	^99^Mo/^99m^Tc < 0.015%	<0.015%	<0.015%	<0.015%	<0.015%
	Other gammas/^99m^Tc < 0.05%	<0.05%	<0.05%	<0.05%	<0.05%
Aluminium	<10 ppm	<10 ppm	<10 ppm	<10 ppm	<10 ppm
Molybdenum	Not specified	—	0.138 ppm	0.020 ppm	0.034 ppm
Radiochemical purity of ^99m^TcO_4_^−^	>95%	99.80%	99.90%	99.59%	99.52%
Radiochemical yield of ^99m^Tc-radiopharmaceuticals	>90%	^99m^Tc-MIBI	^99m^Tc-ECD	^99m^Tc-MAG3	^99m^Tc-MDP
		99.98%	98.27%	97.23%	99.95%

**Table 14.** Recovery yield and distribution of ^100^MoO_3_ after ^99m^Tc thermochromatography and the recovery procedure

	Run 1 (mass/g)	Run 2 (mass/g)
Distribution		

Before separation		
1) Crucibles	129.703	117.490

After separation		

1) Crucibles	118.870	110.551

2) Other than crucibles	10.833	6.939
a) Platinum shelf		0
b) Quartz tubes		0.073
c) Quartz wool		0.376
d) Needle crystal holder	9.477	6.380

Recovery yield		

1) Crucibles	117.502	108.640

2) Other than crucibles (Needle crystal holder)	10.296	8.426

Total	127.798 (98.5%)	117.066 (99.6%)
